# Cross‐Sectional Associations of Body Mass Index With Disability Across High‐ and Middle‐Income Countries in 2002–2006 and 2015–2018

**DOI:** 10.1111/obr.70054

**Published:** 2025-11-30

**Authors:** Marcos D. Machado‐Fragua, Séverine Sabia, Aurore Fayosse, Gabriella C. Silva, Benjamin Landré, Archana Singh‐Manoux

**Affiliations:** ^1^ Epidemiology of Ageing and Neurodegenerative diseases (EpiAgeing) Université Paris Cité, Inserm U1153 Paris France; ^2^ Faculty of Brain Sciences University College London London UK

**Keywords:** ADL limitations, disability, IADL limitations, obesity, sex differences

## Abstract

**Introduction:**

We examined the cross‐sectional association of BMI with limitations in instrumental (IADL) and basic (ADL) activities of daily living in surveys from middle‐ and high‐income countries in 2015–2018; we also compared changes in these associations from 2002–2006 to 2015–2018.

**Methods:**

Data at the 2015–2018 wave were available on 152,856 participants aged ≥ 50 years in seven nationally representative surveys from middle‐ (Mexico, India, and China) and high‐income (the United States, SHARE–European countries, Israel, the United Kingdom, and Korea) countries. BMI was measured or self‐reported, and disability in IADL and ADL was defined as limitations in at least one out of five items, respectively.

**Results:**

The prevalence of underweight in men/women ranged from 0.5/1.5 (UK) to 23.4%/20.6% (India), and that of obesity from 0.7/1.5 (Korea) to 35.6%/37.6% (US), respectively. Meta‐analyses showed underweight to be associated with a higher odds ratio (95% CI) of IADL (men, 1.78[1.26, 2.52]; women 2.07[1.38, 3.10]) and ADL (men, 1.89[1.22, 2.91]; women 1.72[1.16, 2.53]) disability. Obesity was associated with lower IADL limitations among men (0.80 [0.67–0.96]) but not among women (1.18 [0.94–1.49]), and with higher ADL (men, 1.38 [1.14, 1.65]; women, 1.59 [1.37, 1.84]). Associations of underweight with IADL and ADL, and obesity with ADL were stronger in high‐income countries. The association of BMI categories with IADL/ADL was similar in the 2002–2006 data, although the prevalence of obesity was higher in 2015–2018.

**Conclusion:**

Both underweight and obesity are associated with higher IADL and ADL disability; the stronger associations in high‐income countries require further research.

**Abbreviations**
ADL: basic activities of daily living
BMI: body mass index
CHARLS: China Health and Retirement Longitudinal Study
ELSA: English Longitudinal Study of Aging
HIC: high‐income countries
HRS: Health and Retirement Study
IADL: instrumental activities of daily living
KLoSA: Korean Longitudinal Study of Aging
LASI: Longitudinal Aging Study in India
MHAS: Mexican Health and Aging Study
MIC: middle‐income countries
SHARE: Survey of Health, Aging and Retirement in Europe
WHO: World Health Organization

## 1 Introduction

Population aging is accompanied by an increase in the number of people living with functional limitations that hamper their ability to perform everyday activities and their autonomy [1]. There is an urgent need to identify strategies to prevent or delay age‐related disability. Among risk factors for disability, body mass index (BMI) at the extremes, both obesity [2, 3, 4, 5, 6] and underweight [2], has been shown to be associated with a higher risk of limitations in instrumental (IADL) and basic (ADL) activities of daily living. Given the ongoing global obesity epidemic [7], and the persistent prevalence of underweight individuals in some world regions [8], there is interest in understanding whether the relationship between BMI and IADL/ADL limitations varies across countries. This would allow targeted prevention policies to effectively mitigate the burden of BMI‐related disability.

Despite the growing literature on the relationship between BMI and disability, most studies have been conducted in specific countries [2, 3, 6] or specific regions defined by income [5], limiting cross‐income–based comparisons. In addition, there are sex differences in BMI levels across world regions that could result from the cultural context [9]. It is thus important to disentangle the country and sex specificity of the association between BMI and disability, particularly given the findings that the impact of fat distribution on health differs by sex [10, 11, 12]. Finally, temporal trends in the association between BMI and disability, that would be informative in determining whether some countries have managed to reduce the burden of functional limitations associated with extreme BMI categories, remain unclear.

The primary aim of this study was to examine the association of BMI with IADL and ADL limitations across high‐income countries (HIC; United States, several European countries, Israel, and Korea) and middle‐income countries (MIC; Mexico, India, and China) using data on adults over ≥ 50 years from seven nationally representative surveys in 2015–2018, separately in men and women. The secondary aim was to test whether these associations varied between 2002–2006 and 2015–2018.

## 2 Material and Methods

### 2.1 Data Sources

Data were drawn from participants aged ≥ 50 years in 2002–2006 and 2015–2018 (survey years that allowed inclusion of a maximum number of individual studies at two time points) from the US‐based Health and Retirement Study (HRS) [13], the Survey of Health, Aging and Retirement in Europe (SHARE) [14], the English Longitudinal Study of Aging (ELSA) [15], the Korean Longitudinal Study of Aging (KLoSA) [16], the Mexican Health and Aging Study (MHAS) [17], the Longitudinal Aging Study in India (LASI) [18], and the China Health and Retirement Longitudinal Study (CHARLS) [19]. As defined by the World Bank [20], HIC were represented by HRS, SHARE, ELSA, and KLoSA and MIC by MHAS, LASI, and CHARLS. The surveys included in our study were selected based on the age of participants at baseline (≥ 50 years) and the availability of data on BMI and IADL/ADL limitations. We used harmonized data on BMI, IADL/ADL disability, and covariates from “gateway to global aging” (g2aging.org) for comparison between surveys. For the main cross‐sectional analysis of data from 2015 to 2018, the analyses were based on HRS wave 14 (2018), SHARE Wave 7 (2017), ELSA Wave 9 (2018), KLoSA Wave 7 (2018), MHAS Wave 5 (2018), LASI Wave 1 (2017), and CHARLS Wave 3 (2015). For temporal comparison, the data described above were compared with those from 2002 to 2006 from HRS Wave 6 (2002), SHARE Wave 1 (2004), ELSA Wave 2 (2004), KLoSA Wave 1 (2006), and MHAS Wave 2 (2003). Data from the two time periods do not necessarily contain the same individuals, as the studies continue to include new participants over successive waves; the analyses in our study do not imply a follow‐up of the same individuals. The comparisons over time were undertaken only in countries with data at both waves of SHARE (Austria, Germany, Sweden, Spain, Italy, France, Denmark, Greece, Switzerland, Belgium, and Israel).

### 2.2 BMI

Data on weight and height were reported by participants in HRS, SHARE, KLoSA, and MHAS. In ELSA, LASI, and CHARLS, weight and height were measured by trained research staff. BMI was calculated as weight in kilograms divided by the square of the height in meters and categorized as underweight (< 18.5 kg/m^2^), normal weight (≥ 18.5 to < 25 kg/m^2^), overweight (≥ 25 to < 30 kg/m^2^), and obesity (≥ 30 kg/m^2^), using conventional World Health Organization (WHO) thresholds.

### 2.3 ADL and IADL Limitations

Functional limitations were measured using a five‐item IADL scale and a five‐item ADL scale, except MHAS that had a 4‐item IADL scale [21, 22]. Participants were considered to have a limitation on an item if they answered “yes” when asked whether they experienced any difficulty in performing the activity because of a health or memory problem. IADL limitations included difficulties with making telephone calls (not included in the four‐item IADL scale in MHAS), managing money, taking medications, shopping for groceries, and preparing hot meals. ADL limitations included difficulties with bathing, dressing, eating, getting in/out of bed, and using the toilet (controlling urination/defecation in CHARLS). Participants were considered to have limitations if they responded as having limitations on one or more of the items in the IADL (ADL) scales.

### 2.4 Covariates

Covariates included sex (men or women), age (continuous), age^2^ (for nonlinear associations with age), education (low, intermediate, or high), marital status (single; married, or partnered; or divorced, separated, or widowed), and prevalence of diabetes, arthritis, hypertension, stroke, cancer, lung disease, and heart disease.

### 2.5 Statistical Analyses

Missing data on BMI, IADL, ADL, and covariates were imputed using predictive mean matching in multiple imputations with chained equations [23]. The number of imputations was determined using “linear rule of thumb” [23, 24, 25]. The number of imputations was based on 100 minus the percentage of complete cases, plus an additional 10 imputations to be conservative, as determined by the survey with the greatest proportion of missing data. Application of this approach yielded 50 imputations in each survey. Details of the patterns of missingness are provided in Table S1 and of the imputation procedure in Supplementary methods. The analyses used weights, available in each survey, to account for multistage sampling strategies and nonresponse so that estimates from the analyses are nationally representative.

We tested for sex differences using interaction terms between sex and BMI categories, and as all tests had a *p* < 0.05, all analyses were undertaken separately in men and women. In the first analysis, the association between BMI and IADL and then ADL limitations in 2015–2018 was examined separately in each survey using a weighted logistic regression model adjusted for age, age^2^, education, marital status, and prevalence of chronic conditions (analyses in SHARE were further adjusted for country). A random effects meta‐analysis was then used to calculate overall estimates along with the *I* ^2^ test for heterogeneity. To test differences in associations between HIC and MIC we examined subgroup differences using the Q test in the random effects meta‐analyses.

In further analyses, data from surveys in 2002–2006 and 2015–2018 were pooled to determine whether the association of BMI with IADL and ADL limitations varies across the two time periods. These logistic regression models included BMI, age, age^2^, education, marital status, chronic conditions, and the interactions between these variables and the period. *p* values for the interaction between BMI and time period were reported.

To examine the robustness of our results, we first examined whether the association of BMI with IADL and ADL limitations in 2015–2018 varied by age (< 65 years vs. ≥ 65 years) using a logistic regression model adjusted for all covariates. We also repeated the main analyses using Asia‐Pacific WHO thresholds to categorize BMI in the KLoSA, LASI, and CHARLS studies (< 18.5 kg/m^2^ for underweight; ≥ 18.5 to < 23 kg/m^2^ for normal weight; ≥ 23 to < 25 kg/m^2^ for overweight; and ≥ 25 kg/m^2^ for obesity).

In additional analyses, we examined individual IADL and ADL items by fitting separate logistic regression models for each item in the IADL and ADL scales, adjusting for all covariates. Random effects meta‐analyses were then performed to obtain pooled estimates across surveys, and subgroup differences were examined for HIC versus MIC.

Analyses were undertaken using Stata 16.1 (StataCorp). A two‐sided *p* value < 0.05 was considered statistically significant.

## 3 Results

Analyses in 2015–2018 were based on 152,856 respondents aged ≥ 50 years, including 16,069 from HRS, 38,121 from SHARE, 7040 from ELSA, 6940 from KLoSA, 15,858 from MHAS, 52,393 from LASI, and 16,435 from CHARLS (flow chart in Figure S1); corresponding numbers from 2002 to 2006 are also shown in Figure S1. At the 2002–2006 wave, completeness of data varied between 66.9% in MHAS and 97.7% in SHARE; at the 2015–2018 wave, completeness varied between 63.0% in ELSA and 98.5% in KLOSA (Table S1).

Mean BMI in all BMI categories (underweight, normal weight, overweight, and obese) in 2002–2006 and 2015–2018 is shown in Table S2, and the characteristics of the participants at the two time periods are shown in Tables S3 and S4. The prevalence of underweight in 2015–2018 in men ranged from 1.5% (MHAS) to 23.4% (LASI) in MIC, and from 0.5% (ELSA) to 2.4% (KLoSA) in HIC. Estimates in women ranged from 2.1% (MHAS) to 20.6% (LASI), and from 1.5% (ELSA) to 2.9% (KLoSA), respectively (Table 1). The prevalence of underweight decreased between 2002–2006 and 2015–2018 in KLoSA in men, and in HRS and KLoSA in women. The prevalence of obesity in 2015–2018 in men ranged from 3.7% (LASI) to 22.4% (MHAS) in MIC, and from 0.7% (KLoSA) to 35.6% (HRS) in HIC. Corresponding numbers in women ranged from 6.3% (CHARLS) to 31.2% (MHAS), and from 1.5% (KLoSA) to 37.6% (HRS), respectively. The prevalence of obesity increased in both men and women in HRS, SHARE, and MHAS between 2002–2006 and 2015–2018, but it decreased in women in KLoSA.

**TABLE 1:** Change in composition of BMI groups between 2002–2006 and 2015–2018 in men and women.

	BMI categories											
	Underweight			Normal weight			Overweight			Obesity		
	%	%	*p* value^a^	%	%	*p* value^a^	%	%	*p* value^a^	%	%	*p* value^a^
	2002–2006	2015–2018		2002–2006	2015–2018		2002–2006	2015–2018		2002–2006	2015–2018	
Men												
HRS	0.8	0.7	0.54	28.0	19.9	**< 0.01**	45.8	43.8	0.05	25.4	35.6	**< 0.01**
SHARE	0.6	0.7	0.74	33.0	31.5	0.11	50.0	47.6	**0.01**	16.4	20.2	**< 0.01**
ELSA	0.7	0.5	0.37	24.0	24.7	0.70	47.7	45.4	0.16	27.6	29.4	0.38
KLoSA	3.6	2.4	**0.01**	73.6	73.8	0.94	22.0	23.1	0.38	0.8	0.7	0.86
MHAS	1.7	1.5	0.73	37.4	32.6	**0.01**	42.9	43.5	0.78	18.0	22.4	**0.01**
LASI	NA	23.4	NA	NA	55.3	NA	NA	17.6	NA	NA	3.7	NA
CHARLS	NA	6.4	NA	NA	61.4	NA	NA	28.2	NA	NA	4.0	NA
Women												
HRS	2.9	1.9	**< 0.01**	38.1	29.2	**< 0.01**	33.7	31.3	**0.01**	25.3	37.6	**< 0.01**
SHARE	2.1	2.1	0.93	43.3	43.1	0.83	36.5	34.5	**0.01**	18.1	20.3	**< 0.01**
ELSA	1.0	1.5	0.08	28.6	29.9	0.42	39.6	38.0	0.28	30.8	30.6	0.87
KLoSA	4.5	2.9	**< 0.01**	69.2	70.0	0.46	23.8	25.6	0.10	2.5	1.5	**0.01**
MHAS	2.3	2.1	0.84	33.5	26.5	**< 0.01**	39.2	40.2	0.57	25.0	31.2	**< 0.01**
LASI	NA	20.6	NA	NA	48.6	NA	NA	21.7	NA	NA	9.1	NA
CHARLS	NA	6.5	NA	NA	56.5	NA	NA	30.7	NA	NA	6.3	NA

*Note:* Results in the darker shade are for surveys from middle‐income countries. Values in bold are statistically significant.

Abbreviations: BMI: body mass index; CHARLS: China Health and Retirement Longitudinal Study; ELSA: English Longitudinal Study of Aging; HRS: Health and Retirement Study; KLoSA: Korean Longitudinal Study of Aging; LASI: Longitudinal Aging Study in India; MHAS: Mexican Health and Aging Study; SHARE: Survey of Health, Aging and Retirement in Europe.

^a^ *p* value for difference between 2002–2006 and 2015–2018 cross‐sectional data with 2002–2006 being the reference.

**FIGURE 1:**
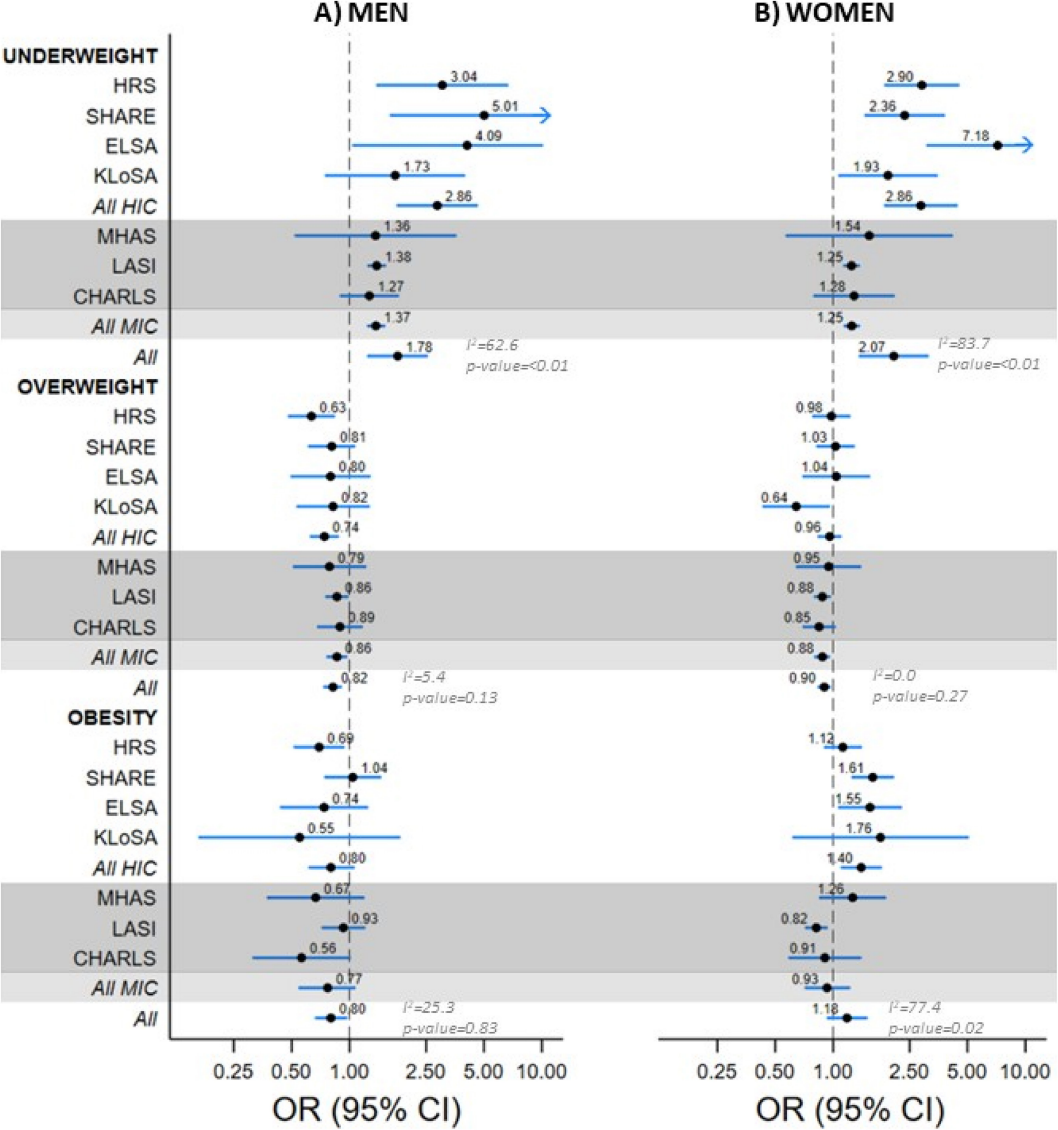
Association between BMI (in categories) and IADL limitations^a^ in men and women using data from 2015–2018^b^. Results in the darker shade are for surveys from middle‐income countries. Abbreviations: BMI: body mass index; IADL: instrumental activities of daily living; HRS: Health and Retirement Study; SHARE: Survey of Health, Ageing and Retirement in Europe; ELSA: English Longitudinal Study of Ageing; KLoSA: Korean Longitudinal Study of Ageing; MHAS: Mexican Health and Aging Study; LASI: Longitudinal Ageing Study in India; CHARLS: China Health and Retirement Longitudinal Study. ^a^Limitations defined as reporting one or more limitations out of five items (four items in the MHAS cohort) on the IADL scale. ^b^Logistic regression model adjusted for age, age^2^, marital status, education, and prevalence of diabetes, arthritis, hypertension, stroke, cancer, lung disease, and heart disease. *p* values for differences in meta‐analysis estimates between HIC and MIC are provided. Arrows denote estimates where the upper limit of the confidence interval exceeds the X‐axis range.

The prevalence of IADL limitations across BMI categories in 2002–2006 and 2015–2018 is shown in Table S5. These data show IADL limitations to be highest in the underweight group in both men and women, at both time periods (2002–2006 and 2015–2018). No significant differences were found between the two time periods in the association between BMI (in categories) and IADL limitations (Table S6). In pooled analyses of all data from 2015 from 2018, being underweight compared with normal weight participants was associated with higher odds ratio (OR) of IADL limitations (OR, 95% CI: 1.78, 1.26–2.52, I^2^ 62.6 in men; 2.07, 1.38–3.10, I^2^ 83.7 in women). Analyses of subgroups (HIC/MIC) suggested differences in associations (*p* values < 0.01 for men and women) with associations in HIC (OR, 95% CI: 2.86, 1.78–4.59 in men; 2.86, 1.87–4.38 in women) being stronger than those in MIC (OR, 95% CI: 1.37, 1.25–1.51, in men; 1.25, 1.15–1.36 in women) (Figure 1 and Table S6). Analyses of individual IADL revealed a consistent pattern, with underweight being associated with higher odds of all limitations in both men and women (all *p* for overall estimates < 0.05; Figure S2).

Among men, obesity was associated with lower odds of IADL limitations (OR, 95% CI: 0.80, 0.67–0.96), with no difference in estimates from HIC and MIC (*p* value = 0.83; Figure 1 and Table S6). This association was mainly due to fewer difficulties in managing money in men with obesity (OR, 95% CI: 0.68, 0.53–0.88; Figure S2). In women, obesity was not associated with IADL limitations in the overall pooled analyses (OR, 95% CI: 1.18, 0.94–1.49), but in HIC, it was associated with higher odds of IADL limitations (OR, 95% CI: 1.40, 1.11–1.77), with a *p* value of 0.02 for the Q test (Figure 1 and Table S6). In women from HIC, these results were mainly due to difficulties in activities such as shopping for groceries (OR, 95% CI: 1.55, 1.32–1.82) and preparing hot meals (OR, 95% CI: 1.25, 1.03–1.53; Figure S2).

**FIGURE 2:**
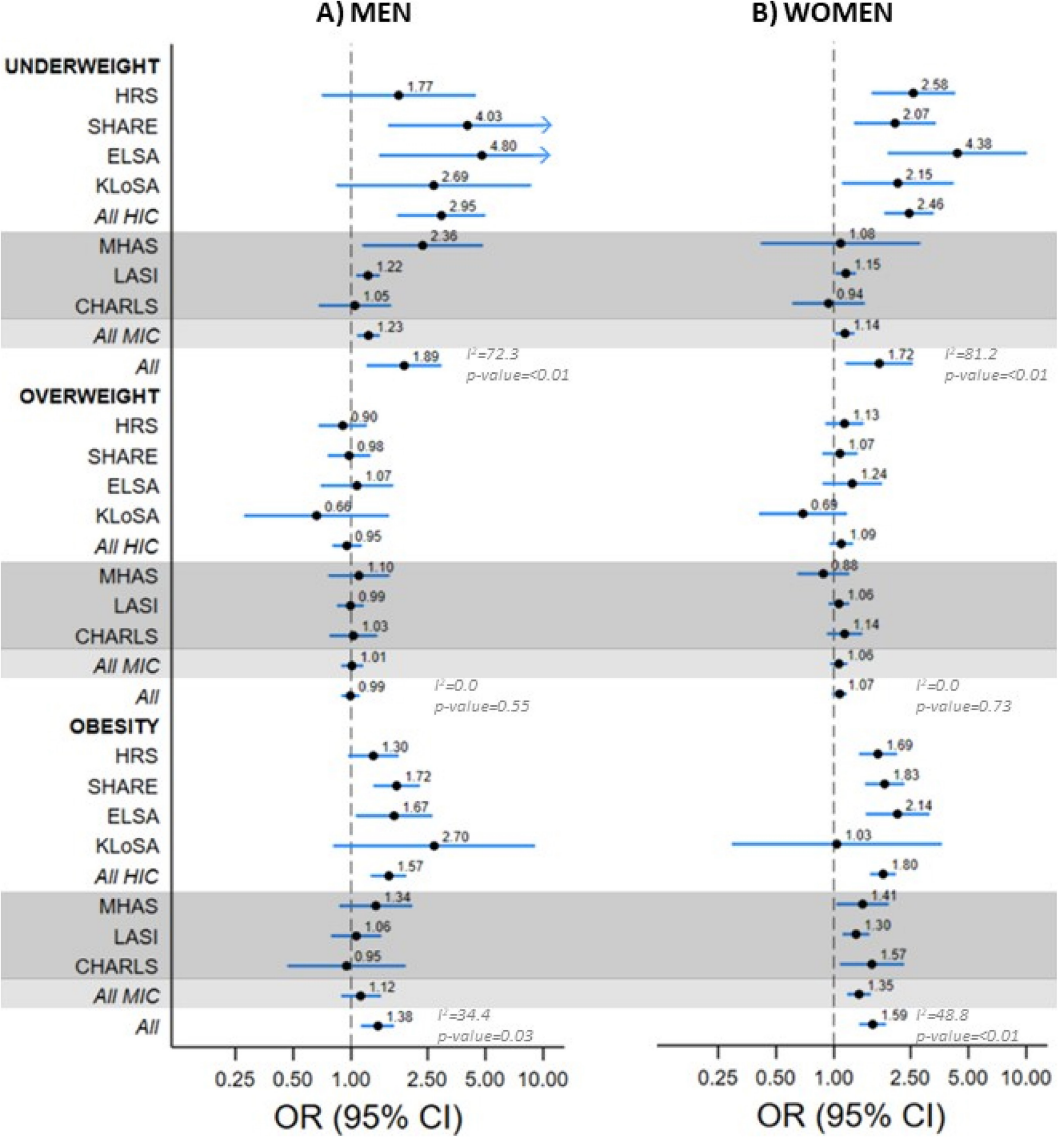
Association between BMI (in categories) and ADL limitations^a^ in men and women using data from 2015–2018^b^. Results in the darker shade are for surveys from middle‐income countries. Abbreviations: BMI: body mass index; ADL: activities of daily living; HRS: Health and Retirement Study; SHARE: Survey of Health, Ageing and Retirement in Europe; ELSA: English Longitudinal Study of Ageing; KLoSA: Korean Longitudinal Study of Ageing; HIC: high‐income countries; MHAS: Mexican Health and Aging Study; LASI: Longitudinal Ageing Study in India; CHARLS: China Health and Retirement Longitudinal Study; MIC: middle‐income countries. ^a^Limitations defined as reporting one or more limitations out of five items on the ADL scale. ^b^Logistic regression model adjusted for age, age^2^, marital status, education, and prevalence of diabetes, arthritis, hypertension, stroke, cancer, lung disease, and heart disease. *p* values for differences in meta‐analysis estimates between HIC and MIC are provided. Arrows denote estimates where the upper limit of the confidence interval exceeds the X‐axis range.

The prevalence of ADL limitations in 2002–2006 and 2015–2018 was higher in the extreme BMI categories (underweight and obesity) in both men and women, except CHARLS where obesity in men had the lowest level of ADL limitations compared with the other BMI categories (Table S7). The results of logistic regression, using the normal weight group as the reference, for the association between BMI and ADL limitations in both time periods are shown in Figure 2 and Table S8. Only in ELSA men and women was there evidence of a stronger association between BMI and ADL limitations over time. Men and women in the underweight group in 2015–2018 had higher odds of ADL limitations in the pooled analyses (OR, 95% CI: 1.89, 1.22–2.91 in men; and 1.72, 1.16–2.53 in women) that were characterized by considerable heterogeneity (I^2^ = 72.3% in men, I^2^ = 81.2% in women). Analyses for subgroup differences (*p* values < 0.01 in men and women) suggested stronger associations in HIC (OR, 95% CI: 2.95, 1.76–4.93 in men; and 2.46, 1.85–3.26 in women) than MIC (OR, 95% CI: 1.23, 1.09–1.39 in men; and 1.14, 1.03–1.26 in women). In analyses of individual ADL items, underweight was consistently associated with higher odds of all limitations in both men and women (all *p* for overall estimates < 0.05; Figure S3).

At the 2015–2018 wave, obesity in men was associated with higher odds of ADL limitations in the pooled analyses (OR, 95% CI: 1.38, 1.14–1.65) with some heterogeneity (I^2^ = 34.4%) reflected in associations in HIC (OR, 95% CI: 1.57, 1.28–1.91) but not MIC (OR, 95% CI: 1.12, 0.90–1.41), with Q‐test showing differences between the two groups (*p* value = 0.03) (Figure 2 and Table S8). These results in men were mainly due to higher odds of difficulty in dressing (OR, 95% CI: 1.59, 1.29–1.97; Figure S3). In women, obesity was linked to higher odds of ADL limitations overall (OR, 95% CI: 1.59, 1.37–1.84; I^2^ = 48.8%), with associations being stronger (*p* value < 0.01) in HIC (OR, 95% CI: 1.80, 1.56–2.07) than MIC (OR, 95% CI: 1.35, 1.19–1.53). These associations were primarily driven by higher odds of difficulty in bathing (OR, 95% CI: 1.42, 1.13–1.79), dressing (OR, 95% CI: 1.68, 1.32–2.15), getting in/out of bed (OR, 95% CI: 1.41, 1.24–1.59), and using the toilet (OR, 95% CI: 1.41, 1.25–1.60; Figure S3).

Additional analyses using the 2015–2018 data to examine whether age modified the association between BMI and IADL/ADL limitations suggested no differences in association in those < 65 years or ≥ 65 years in men and women (Table S9). Analyses using Asia‐Pacific WHO thresholds to categorize BMI in KLoSA, LASI, and CHARLS yielded similar results to those in the main analysis, with the direction of the associations remaining unchanged for overall and MIC estimates (Figures S4 and S5), but differences between HIC and MIC in the association of obesity with ADL limitations in women were attenuated (*p* value = 0.21; Figure S5).

## 4 Discussion

This study examined the cross‐sectional associations of BMI with IADL and ADL limitations during two time periods, separated by 12 years, in seven nationally representative studies representing MIC and HIC. There was some change over time in the composition of BMI groups, characterized by a reduction in underweight and an increase in obesity but associations of BMI with IADL and ADL remained similar across the time period. We found underweight to be associated with a higher prevalence of IADL limitations in men and women but obesity was related to a lower prevalence of IADL limitations in men. Both underweight and obesity were associated with a higher prevalence of ADL limitations in men and women, with associations stronger in HIC rather than MIC.

Previous research has shown sex differences in the distribution of BMI categories and in the prevalence of IADL/ADL limitations [26, 27, 28]. Women have been reported to have higher rates of both obesity and disability [5, 29, 30, 31, 32]. The rates of underweight and obesity in 2015–2018 in our study were generally higher among women than among men. The rates of underweight were highest in LASI, the survey from India, at 23.4% in men and 20.6% in women. The rates of underweight in HIC in men varied between 0.5% and 2.4%, and in women between 1.5% and 2.9%. The prevalence of obesity was highest in HRS, the survey from the US, where it was 35.6% in men and 37.6% in women. There were some changes over time in BMI, the most remarkable being an increase in obesity in HRS, from 25.4% in 2002–2006 to 35.6% in 2015–2018 in men, the corresponding numbers being 25.3% and 37.6% in women.

We found sex differences in the association of BMI with IADL/ADL, leading us to undertake analyses separately in men and women. These differences are likely to be due to a range of biological, behavioral, hormonal, and social factors. Women typically have higher body fat with higher fat levels in hips and thighs, that may affect mobility and physical function differently than men, where there is higher accumulation of visceral fat [10, 11]. In addition, hormonal factors in women contribute to the preservation of muscle mass and avoidance of visceral fat deposition, but this protective effect dissipates after menopause, leading to higher levels of disability in women [11, 12]. Women have greater mobility limitations than men, starting in midlife [33], that may also affect functional limitations such as the ADL items.

Our findings are consistent with previous research showing stronger associations between BMI and disability in women [2, 4, 5, 6, 34]. To better understand sex‐specific associations in our study, we undertook additional analyses on individual IADL and ADL items. These analyses showed underweight to be consistently associated with higher odds of difficulties across all individual IADL and ADL limitations in both men and women, likely due to frailty and reduced physiological reserve in underweight individuals.

The impact of obesity on IADL in our study was more nuanced. The lower odds of IADL in men with obesity were primarily driven by better performance in managing money rather than other items in IADL. In women with obesity, particularly in HIC, mobility‐dependent activities (shopping for groceries and preparing hot meals) were responsible for the higher odds of IADL; cognitively demanding tasks (managing money, making telephone calls, and taking medication) were not affected. This pattern in HIC likely reflects higher education, greater independence, and birth cohort‐specific historical gender roles, which enabled women to perform cognitively complex tasks. This pattern was similar for cognitively demanding IADL (making telephone calls, managing money, and taking medications) in MIC but no associations were found with mobility‐related IADL, possibly because many women do not perform these tasks independently and rely on family or community support [22, 35, 36].

Obesity impacted ADL in both men and women; in men, it was due to difficulties with dressing but in women the impact was more extensive (bathing, dressing, getting in/out of bed, and using the toilet). These results highlight that mobility‐related limitations are more common in women. Previous research has shown ADL to primarily capture mobility and self‐care functions, while IADL reflects more cognitively demanding tasks (handling finances, using the telephone, and managing medications) [22, 37, 38, 39]. These findings highlight the importance of mobility‐related issues in women with obesity. Contextual factors are likely to explain observed differences between HIC and MIC in women.

Most previous studies on the impact of BMI on functional limitations have been undertaken in individual countries [2, 3, 6] or within specific income regions [5]. The exception is a previous cross‐sectional study on adults aged ≥ 50 years from nine low‐, MIC, and HIC that reported a stronger association between obesity and ADL limitations in HIC compared with low‐income and MIC [40]. Our results are consistent with these findings; we were able to add to this research by examining individual items in ADL. We also extend these findings by including IADL in our analyses, and we found obesity in women to have stronger associations with IADL in HIC. Importantly, a novel finding of our study is that underweight shows a stronger association with both IADL and ADL limitations in HIC for men and women.

In general terms, the prevalence of IADL and ADL as a function of BMI categories was more marked in HIC than MIC. These differences highlight the complex interplay of health, economic, social, and cultural factors in determining how BMI affects daily functioning [41, 42, 43]. The stronger associations found between extreme BMI categories (underweight and obesity) and disability, measured using IADL and ADL, in HIC may be due to several factors such as more sedentary lifestyles in HIC, and selective survival bias in MIC data. In our study, HIC participants were somewhat older (mean age ranged from 64.6 to 66.0) than MIC (mean age ranged from 61.8 to 63.8) participants. In addition, the prevalence of chronic conditions, such as cardiovascular diseases and diabetes, in underweight and obesity groups was generally higher in HIC as compared with MIC. These conditions may be more effectively managed in HIC than in MIC [44], resulting in longer but potentially more disabled lives in HIC, leading to weaker associations between BMI and functional limitations in MIC. Additionally, sedentary lifestyles in HIC may exacerbate functional limitations associated with these conditions [45]. Moreover, cohort effects, historical gender roles, and patterns of task distribution are likely to contribute to the observed sex‐ and context‐specific differences [36]. Our findings emphasize the importance of considering the income level of a country and contextual factors when designing preventive strategies to mitigate BMI‐related disability.

Examining changes in the trend of the association between BMI and disability over time is essential for understanding how shifts in population health, advancements in medical care, and changes in lifestyle impact functional outcomes in aging populations. Additionally, it allows the evaluation of long‐term effects of health policies and demographic differences, providing critical insights for reducing the disability burden and improving the quality of life among older adults. We found changes in the composition of BMI categories, primarily an increase in obesity rates, but no differences in the association between BMI and IADL/ADL disability over time. Previous research using data from the National Health and Nutrition Examination Survey (NHANES) in the US suggested an increase in the odds of disability among individuals with obesity between 1988–1994 and 1999–2004 [46, 47]. However, this increase was attenuated when data up to 2012 were analyzed [47]. The absence of changes in the trend of the association between BMI and disability over time may indicate that the underlying relationship is stable, but the increase in obesity levels suggests that a greater number of people will have functional limitations.

This study on the association between BMI and IADL/ADL limitations across MIC and HIC has several strengths. We used data from seven nationally representative surveys to account for variations between HIC and MIC, and considered two time periods to understand variations in the relationship over time. However, this study should be interpreted in light of its limitations. One, our analyses were cross‐sectional, not allowing conclusions to be drawn on the causal nature of the association between BMI and IADL/ADL limitations. Two, IADL/ADL limitations as well as BMI, in several surveys, were self‐reported and may be subject to reporting bias. Self‐reported measures are dependent on personal perception, and this may be conditional on several factors such as sex and country characteristics. Three, thresholds to define BMI categories may be race‐specific, complicating cross‐national comparisons. However, we performed sensitivity analyses using Asia‐Pacific thresholds for BMI in KLoSA, CHARLS, and LASI, and associations remained unchanged. Four, harmonized measures of cognitive function were not available across all studies and could not be used as a covariate in the analyses. Nevertheless, our analyses of individual IADL/ADL items suggest that the observed associations are more likely driven by mobility‐related limitations rather than cognitive status. Five, as in all observation studies residual confounding may persist despite careful adjustment for covariates in our analyses.

## 5 Conclusion

Underweight and obesity are significant threats to health and carry a substantial economic burden on healthcare systems. Our findings suggest that these associations of BMI with IADL/ADL are activity‐specific and sex‐specific, with mobility‐related tasks being affected in particular. Analyses of individual IADL and ADL items provide actionable insights that can inform population‐ and individual‐level interventions tailored to sex, BMI category, and functional domain. Promotion of healthy body weight is paramount in mitigating BMI‐related comorbidity, particularly for women and for individuals in HIC, where these associations are more pronounced despite advances in health care.

## Funding

This study was supported by Agence Nationale de la Recherche, France 2030 ANR‐23‐PAVH‐0006.

## Ethics Statement

Ethical approval for HRS was obtained from the University of Michigan Institutional Review Board and the study has been conducted according to the principles expressed in the Declaration of Helsinki.

The SHARE study is subject to continuous ethics review. The continuation of the project was reviewed and approved by the Ethics Council of the Max‐Planck‐Society.

The survey of the Korean Longitudinal Study of Aging (KLoSA) was approved by the Institutional Review Board of the Korea Centers for Disease Control and Prevention.

The MHAS study protocol and instruments were approved by the Institutional Review Board or Ethics Committee of the University of Texas Medical Branch, the INEGI in Mexico, and the Instituto Nacional de Salud Pública (INSP) in Mexico.

For LASI, ethical approvals were obtained from the following collaborating organizations: Indian Council of Medical Research (ICMR), Delhi; IRB, International Institute for Population Sciences (IIPS), Mumbai; IRB, Harvard T.H. Chan School of Public Health (HSPH), Boston; IRB, University of Southern California (USC), Los Angeles; IRB, ICMR‐National AIDS Research Institute (NARI), Pune; and IRB, Regional Geriatric Centres (RGCs), MoHFW.

CHARLS was approved by the Ethical Review Committee of Beijing University, and all participants signed informed consent at the time of participation.

All participants signed an informed consent form before the interviews. No additional ethical approval is needed for the present study.

## Conflicts of Interest

The authors declare no conflicts of interest.

## Supporting information


**Table S1:** Data completeness and missing patterns in the surveys included in the analyses.
**Table S2:** Mean BMI across BMI categories in men and women at the 2002–2006 and 2015–2018 waves of data collection.
**Table S3:** Characteristics of men and women from the 2002–2006 wave, after imputation for missing data and weighting to obtain national representative data.
**Table S4:** Characteristics of men and women from the 2015 to 2018 wave, after imputation for missing data and weighting to obtain national representative data.
**Table S5:** Prevalence of IADL limitations, weighted to be nationally representative.
**Table S6:** The odds ratio of IADL limitations in underweight, overweight, and obesity groups compared with normal weight men and women in cross‐sectional analyses using data from 2002–2006 to 2015–2018.
**Table S7:** Prevalence of ADL limitations, weighted to be nationally representative.
**Table S8:** The odds ratio of ADL limitations in underweight, overweight, and obesity groups compared with normal weight men and women in cross‐sectional analyses using data from 2002–2006 to 2015–2018.
**Table S9:** *p* values of the test for interaction to examine whether age (< 65 years vs. ≥ 65 years) modifies the association between BMI and IADL/ADL limitations in men and women (2015–2018).
**Figure S1:** Flow chart of sample selection.
Figure S2:*. Association between BMI and individual IADL limitations^a^ in men and women using data from 2015 to 2018.^b^
Figure S3:*. Association between BMI and individual ADL limitations^a^ in men and women using data from 2015 to 2018.^b^
**Figure S4:** Association between BMI (using alternative WHO thresholds for Asia‐Pacific countries) and IADL limitations in men and women using data from 2015 to 2018.
**Figure S5:** Association between BMI (using alternative WHO thresholds for Asia‐Pacific countries) and ADL limitations in men and women using data from 2015 to 2018.

## Data Availability

The HRS (Health and Retirement Study) is sponsored by the National Institute on Aging (grant number NIA U01AG009740) and is conducted by the University of Michigan. HRS data are accessible after registration at (https://hrsdata.isr.umich.edu/data‐products/rand). Survey of Health, Aging and Retirement in Europe (SHARE) data are accessible after registration with the SHARE project at the following addresses: Wave 1 (DOI:10.6103/SHARE.w1.710) and Wave 7 (DOI:10.6103/SHARE.w7.711). English Longitudinal Study of Aging data is freely available to researchers through the UK data service. KLoSA data used in this study is available after registration at https://survey.keis.or.kr/eng/klosa/databoard/List.jsp. MHAS data are available after registration at http://www.mhasweb.org/. LASI data are available upon applying for access at the Gateway to Global Aging platform (https://g2aging.org/). CHARLS data are available after registration at http://charls.pku.edu.cn/en.

## References

[1] J. R. Beard , A. Officer , I. A. de Carvalho , et al., “The World Report on Ageing and Health: A Policy Framework for Healthy Ageing,” Lancet 387 (2016): 2145–2154. 26520231 10.1016/S0140-6736(15)00516-4 PMC4848186

[2] S. Larrieu , K. Pérès , L. Letenneur , et al., “Relationship Between Body Mass Index and Different Domains of Disability in Older Persons: The 3C Study,” International Journal of Obesity and Related Metabolic Disorders 28 (2004): 1555–1560. 15314627 10.1038/sj.ijo.0802755

[3] A. Peeters , L. Bonneux , W. J. Nusselder , C. De Laet , and J. J. Barendregt , “Adult Obesity and the Burden of Disability Throughout Life,” Obesity Research 12 (2004): 1145–1151. 15292479 10.1038/oby.2004.143

[4] H. Chen and X. Guo , “Obesity and Functional Disability in Elderly Americans,” Journal of the American Geriatrics Society 56 (2008): 689–694. 18266843 10.1111/j.1532-5415.2007.01624.x PMC2391089

[5] S. Al Snih , J. E. Graham , Y. F. Kuo , J. S. Goodwin , K. S. Markides , and K. J. Ottenbacher , “Obesity and Disability: Relation Among Older Adults Living in Latin America and the Caribbean,” American Journal of Epidemiology 171 (2010): 1282–1288. 20472569 10.1093/aje/kwq087 PMC2915495

[6] K. Backholer , K. Pasupathi , E. Wong , A. Hodge , C. Stevenson , and A. Peeters , “The Relationship Between Body Mass Index Prior to Old Age and Disability in Old Age,” International Journal of Obesity 36 (2012): 1180–1186. 22732910 10.1038/ijo.2012.99

[7] I. Lingvay , R. V. Cohen , C. W. L. Roux , and P. Sumithran , “Obesity in Adults,” Lancet 404 (2024): 972–987. 39159652 10.1016/S0140-6736(24)01210-8

[8] NCD Risk Factor Collaboration (NCD‐RisC) , “Worldwide Trends in Underweight and Obesity From 1990 to 2022: A Pooled Analysis of 3663 Population‐Representative Studies With 222 Million Children, Adolescents, and Adults,” Lancet 403, no. 10431 (2024): 1027–1050. 38432237 10.1016/S0140-6736(23)02750-2 PMC7615769

[9] C. Koliaki , M. Dalamaga , and S. Liatis , “Update on the Obesity Epidemic: After the Sudden Rise, Is the Upward Trajectory Beginning to Flatten?,” Current Obesity Reports 12 (2023): 514–527. 37779155 10.1007/s13679-023-00527-y PMC10748771

[10] K. Karastergiou , S. R. Smith , A. S. Greenberg , and S. K. Fried , “Sex Differences in Human Adipose Tissues—The Biology of Pear Shape,” Biology of Sex Differences 3 (2012): 13. 22651247 10.1186/2042-6410-3-13 PMC3411490

[11] M. L. Power and J. Schulkin , “Sex Differences in Fat Storage, Fat Metabolism, and the Health Risks From Obesity: Possible Evolutionary Origins,” British Journal of Nutrition 99 (2008): 931–940. 17977473 10.1017/S0007114507853347

[12] M. L. Maltais , J. Desroches , and I. J. Dionne , “Changes in Muscle Mass and Strength After Menopause,” Journal of Musculoskeletal & Neuronal Interactions 9 (2009): 186–197. 19949277

[13] A. Sonnega , J. D. Faul , M. B. Ofstedal , K. M. Langa , J. W. Phillips , and D. R. Weir , “Cohort Profile: The Health and Retirement Study (HRS),” International Journal of Epidemiology 43 (2014): 576–585. 24671021 10.1093/ije/dyu067 PMC3997380

[14] A. Börsch‐Supan , M. Brandt , C. Hunkler , et al., “Data Resource Profile: The Survey of Health, Ageing and Retirement in Europe (SHARE),” International Journal of Epidemiology 42 (2013): 992–1001. 23778574 10.1093/ije/dyt088 PMC3780997

[15] A. Steptoe , E. Breeze , J. Banks , and J. Nazroo , “Cohort Profile: The English Longitudinal Study of Ageing,” International Journal of Epidemiology 42 (2013): 1640–1648. 23143611 10.1093/ije/dys168 PMC3900867

[16] Service KEI , Korea Employment Information Service. KLoSA (Korean Longitudinal Study of Aging), (2018).

[17] R. Wong , A. Michaels‐Obregon , and A. Palloni , “Cohort Profile: The Mexican Health and Aging Study (MHAS),” International Journal of Epidemiology 46 (2017): e2. 25626437 10.1093/ije/dyu263 PMC5837398

[18] International Institute for Population Sciences N , MoHFW , Harvard T. H . Chan School of Public Health, and University of California, Los Angeles, Longitudinal Ageing Study in India (LASI) Wave 1, 2017–18, India Report, (2020).

[19] Y. Zhao , Y. Hu , J. P. Smith , J. Strauss , and G. Yang , “Cohort Profile: The China Health and Retirement Longitudinal Study (CHARLS),” International Journal of Epidemiology 43 (2014): 61–68. 23243115 10.1093/ije/dys203 PMC3937970

[20] World Bank , “World Bank Country and Lending Groups,” The World Bank, (2020).

[21] S. Katz , A. B. Ford , R. W. Moskowitz , B. A. Jackson , and M. W. Jaffe , “Studies of Illness in the Aged: The Index of ADL: A Standardized Measure of Biological and Psychosocial Function,” Journal of the American Medical Association 185 (1963): 914–919. 14044222 10.1001/jama.1963.03060120024016

[22] M. P. Lawton and E. M. Brody , “Assessment of Older People: Self‐Maintaining and Instrumental Activities of Daily Living1,” Gerontologist 9 (1969): 179–186. 5349366

[23] M. Bloomberg , A. Dugravot , A. Sommerlad , M. Kivimäki , A. Singh‐Manoux , and S. Sabia , “Comparison of Sex Differences in Cognitive Function in Older Adults Between High‐ and Middle‐Income Countries and the Role of Education: A Population‐Based Multicohort Study,” Age and Ageing 52 (2023): afad019. 36821646 10.1093/ageing/afad019 PMC9949595

[24] S. van Buuren , Flexible Imputation of Missing Data, Second edition ed., (Chapman and Hall/CRC, 2018).

[25] T. E. Bodner , “What Improves With Increased Missing Data Imputations?,” Structural Equation Modeling: A Multidisciplinary Journal 15 (2008): 651–675.

[26] L. L. Scheel‐Hincke , S. Möller , R. Lindahl‐Jacobsen , B. Jeune , and L. J. Ahrenfeldt , “Cross‐National Comparison of Sex Differences in ADL and IADL in Europe: Findings From SHARE,” European Journal of Ageing 17 (2020): 69–79. 32158373 10.1007/s10433-019-00524-y PMC7040124

[27] M. Bloomberg , A. Dugravot , B. Landré , et al., “Sex Differences in Functional Limitations and the Role of Socioeconomic Factors: A Multi‐Cohort Analysis,” Lancet Healthy Longevity 2 (2021): e780–e790. 34901907 10.1016/S2666-7568(21)00249-X PMC8636280

[28] G. Muscogiuri , L. Verde , C. Vetrani , L. Barrea , S. Savastano , and A. Colao , “Obesity: A Gender‐View,” Journal of Endocrinological Investigation 47 (2024): 299–306. 37740888 10.1007/s40618-023-02196-z PMC10859324

[29] F. C. Drumond Andrade , A. I. Mohd Nazan , M. L. Lebrão , and Y. A. de Oliveira Duarte , “The Impact of Body Mass Index and Weight Changes on Disability Transitions and Mortality in Brazilian Older Adults,” Journal of Aging Research 2013 (2013): 905094. 23691319 10.1155/2013/905094 PMC3649191

[30] B. S. Armour , E. Courtney‐Long , V. A. Campbell , and H. R. Wethington , “Estimating Disability Prevalence Among Adults by Body Mass Index: 2003–2009 National Health Interview Survey,” Preventing Chronic Disease 9 (2012): E178 quiz E78. 23270667 10.5888/pcd9.120136 PMC3534133

[31] Y. M. Na , H. A. Park , J. H. Kang , et al., “Obesity, Obesity Related Disease, and Disability,” Korean Journal of Family Medicine 32 (2011): 412–422. 22745880 10.4082/kjfm.2011.32.7.412 PMC3383150

[32] S. B. Angleman , T. B. Harris , and D. Melzer , “The Role of Waist Circumference in Predicting Disability in Periretirement Age Adults,” International Journal of Obesity 30 (2006): 364–373. 16231023 10.1038/sj.ijo.0803130

[33] M. Bloomberg , A. Dugravot , B. Landre , et al., “Sex Differences in Functional Limitations and the Role of Socioeconomic Factors: A Multi‐Cohort Analysis,” Lancet Healthy Longevity 2 (2021): e780–e790. 34901907 10.1016/S2666-7568(21)00249-X PMC8636280

[34] J. M. Friedmann , T. Elasy , and G. L. Jensen , “The Relationship Between Body Mass Index and Self‐Reported Functional Limitation Among Older Adults: A Gender Difference,” Journal of the American Geriatrics Society 49 (2001): 398–403. 11347782 10.1046/j.1532-5415.2001.49082.x

[35] C. Collingwood , S. M. Paddick , A. Kisoli , et al., “Development and Community‐Based Validation of the IDEA Study Instrumental Activities of Daily Living (IDEA‐IADL) Questionnaire,” Global Health Action 7 (2014): 25988. 25537940 10.3402/gha.v7.25988 PMC4275650

[36] P. S. Mathuranath , A. George , P. J. Cherian , R. Mathew , and P. S. Sarma , “Instrumental Activities of Daily Living Scale for Dementia Screening in Elderly People,” International Psychogeriatrics 17 (2005): 461–474. 16252378 10.1017/s1041610205001547

[37] T. Kekäläinen , M. Luchetti , A. Sutin , and A. Terracciano , “Functional Capacity and Difficulties in Activities of Daily Living From a Cross‐National Perspective,” Journal of Aging and Health 35 (2023): 356–369. 36245236 10.1177/08982643221128929 PMC10104963

[38] D. O. Clark , T. E. Stump , and F. D. Wolinsky , “A Race‐ and Gender‐Specific Replication of Five Dimensions of Functional Limitation and Disability,” Journal of Aging and Health 9 (1997): 28–42. 10182409 10.1177/089826439700900102

[39] R. McGrath , B. M. Vincent , K. J. Hackney , et al., “Weakness and Cognitive Impairment Are Independently and Jointly Associated With Functional Decline in Aging Americans,” Aging Clinical and Experimental Research 32 (2020): 1723–1730. 31520335 10.1007/s40520-019-01351-y

[40] A. Koyanagi , M. V. Moneta , N. Garin , et al., “The Association Between Obesity and Severe Disability Among Adults Aged 50 or Over in Nine High‐Income, Middle‐Income and Low‐Income Countries: A Cross‐Sectional Study,” BMJ Open 5 (2015): e007313. 10.1136/bmjopen-2014-007313 PMC4390733 25838510

[41] J. P. Mackenbach , J. R. Valverde , B. Artnik , et al., “Trends in Health Inequalities in 27 European Countries,” National Academy of Sciences of the United States of America 115 (2018): 6440–6445. 10.1073/pnas.1800028115 PMC6016814 29866829

[42] M. Devaux and F. Sassi , “Social Inequalities in Obesity and Overweight in 11 OECD Countries,” European Journal of Public Health 23 (2011): 464–469. 21646363 10.1093/eurpub/ckr058

[43] B. M. Popkin , L. S. Adair , and S. W. Ng , “Global Nutrition Transition and the Pandemic of Obesity in Developing Countries,” Nutrition Reviews 70 (2012): 3–21. 22221213 10.1111/j.1753-4887.2011.00456.x PMC3257829

[44] D. A. Watkins , W. T. Msemburi , S. J. Pickersgill , et al., “NCD Countdown 2030: Efficient Pathways and Strategic Investments to Accelerate Progress Towards the Sustainable Development Goal Target 3.4 in Low‐Income and Middle‐Income Countries,” Lancet 399 (2022): 1266–1278. 35339227 10.1016/S0140-6736(21)02347-3 PMC8947779

[45] U. Ekelund , J. Steene‐Johannessen , W. J. Brown , et al., “Does Physical Activity Attenuate, or Even Eliminate, the Detrimental Association of Sitting Time With Mortality? A Harmonised Meta‐Analysis of Data From More Than 1 Million Men and Women,” Lancet 388 (2016): 1302–1310. 27475271 10.1016/S0140-6736(16)30370-1

[46] D. E. Alley and V. W. Chang , “The Changing Relationship of Obesity and Disability, 1988–2004,” Journal of the American Medical Association 298 (2007): 2020–2027. 17986695 10.1001/jama.298.17.2020

[47] V. W. Chang , D. E. Alley , and J. B. Dowd , “Trends in the Relationship Between Obesity and Disability, 1988–2012,” American Journal of Epidemiology 186 (2017): 688–695. 28486588 10.1093/aje/kwx092

