# Supplementary material for: Cross‐Sectional Associations of Body Mass Index With Disability Across High‐ and Middle‐Income Countries in 2002–2006 and 2015–2018
Source: Obes Rev. 2025 Nov 30;27(4):e70054. doi: 10.1111/obr.70054 (PMC13008599; doi:10.1111/obr.70054)
Supplement: Supplementary file 1 — Table S1: Data completeness and missing patterns in the surveys included in the analyses. Table S2: Mean BMI across BMI categories in men and women at the 2002–2006 and 2015–2018 waves of data collection. Table S3: Characteristics of men and women from the 2002–2006 wave, after imputation for missing data and weighting to obtain national representative data. Table S4: Characteristics of men and women from the 2015 to 2018 wave, after imputation for missing data and weighting to obtain national representative data. Table S5: Prevalence of IADL limitations, weighted to be nationally representative. Table S6: The odds ratio of IADL limitations in underweight, overweight, and obesity groups compared with normal weight men and women in cross‐sectional analyses using data from 2002–2006 to 2015–2018. Table S7: Prevalence of ADL limitations, weighted to be nationally representative. Table S8: The odds ratio of ADL limitations in underweight, overweight, and obesity groups compared with normal weight men and women in cross‐sectional analyses using data from 2002–2006 to 2015–2018. Table S9: p values of the test for interaction to examine whether age (< 65 years vs. ≥ 65 years) modifies the association between BMI and IADL/ADL limitations in men and women (2015–2018). Figure S1: Flow chart of sample selection. Figure S2:*. Association between BMI and individual IADL limitationsa in men and women using data from 2015 to 2018.b Figure S3:*. Association between BMI and individual ADL limitationsa in men and women using data from 2015 to 2018.b Figure S4: Association between BMI (using alternative WHO thresholds for Asia‐Pacific countries) and IADL limitations in men and women using data from 2015 to 2018. Figure S5: Association between BMI (using alternative WHO thresholds for Asia‐Pacific countries) and ADL limitations in men and women using data from 2015 to 2018. [file OBR-27-e70054-s001.pdf]

Cross-sectional associations of body mass index with disability across high- and middle-income countries in 2002-2006 and 2015-2018

Marcos D. Machado-Fragua PhD[1*]

Séverine Sabia PhD[1,2]

Aurore Fayosse MSc[1]

Gabriella C Silva PhD[1]

Benjamin Landré[1]

Archana Singh-Manoux PhD[1,2]

[1]Université Paris Cité, Inserm U1153, Epidemiology of Ageing and Neurodegenerative diseases (EpiAgeing), Paris, France

[2]Faculty of Brain Sciences, University College London, UK

*Address for correspondence
Université Paris Cité
Inserm U1153, Epidemiology of Ageing and Neurodegenerative diseases
10 Avenue de Verdun, 75010 Paris, France
Tel: +33 (0) 157279045
Email: marcos.machado@inserm.fr
Twitter: @machadofragua ; @epiageing

SUPPLEMENTARY MATERIAL

Supplementary methods

Table S1. Data completeness and missing patterns in the surveys included in the analyses.

Table S2. Mean BMI across BMI categories in men and women at the 2002-2006 and 2015-2018 waves of data collection.

Table S3. Characteristics of men and women from the 2002-2006 wave, after imputation for missing data and weighting to obtain national representative data.

Table S4. Characteristics of men and women from the 2015-2018 wave, after imputation for missing data and weighting to obtain national representative data.

Table S5. Prevalence of IADL limitations, weighted to be nationally representative.

Table S6. The odds ratio of IADL limitations in underweight, overweight, and obesity groups compared to normal weight men and women in cross-sectional analyses using data from 2002-2006 and 2015-2018.

Table S7. Prevalence of ADL limitations, weighted to be nationally representative.

Table S8. The odds ratio of ADL limitations in underweight, overweight, and obesity groups compared to normal weight men and women in cross-sectional analyses using data from 2002-2006 and 2015-2018.

Table S9. P-values of the test for interaction to examine whether age (<65 years vs ≥65 years) modifies the association between BMI and IADL/ADL limitations in men and women (2015-2018).

Figure S1. Flow chart of sample selection

Figure S2*. Association between BMI and individual IADL limitations[a] in men and women using data from 2015-2018.[b]

Figure S3*. Association between BMI and individual ADL limitations[a] in men and women using data from 2015-2018.[b]

Figure S4. Association between BMI (using alternative WHO thresholds for Asia-Pacific countries) and IADL limitations in men and women using data from 2015-2018.

Figure S5. Association between BMI (using alternative WHO thresholds for Asia-Pacific countries) and ADL limitations in men and women using data from 2015-2018.

Supplementary methods

Imputation of missing data

The variables included in the imputation model were: 1) BMI, age, age^2, sex, marital status, education, country in SHARE, prevalence of chronic conditions, and interactions of BMI with age and age^2; 2) auxiliary variables (not used in the main analysis but informative for the missingness pattern): labour force status (employed, unemployed, retired, homemaker); and 3) survey weights, including interaction terms between survey weights and each variable in the imputation model in order to correctly specify the imputation model.[1]

1. Bloomberg M, Dugravot A, Sommerlad A, Kivimaki M, Singh-Manoux A, Sabia S: Comparison of sex differences in cognitive function in older adults between high- and middle-income countries and the role of education: a population-based multicohort study. Age Ageing 2023, 52(2).

Table S1.* Data completeness and missing patterns in the surveys included in the analyses.

Survey, year (Total N, % complete cases)	The number (%) of imputed data													
	Exposure	Outcomes[a]		Covariates										
								Comorbidities						
	BMI	IADL	ADL	Age	Sex	Marital status	Education	Diabetes	Arthritis	Hyper-tension	Stroke	Cancer	Lung disease	Heart disease
**2002-2006 cross-sectional analysis**														
**HRS, 2002** (16720, 96.9)	336 (2.0)	8 (0.0)	7 (0.0)	513 (3.1)	0 (0.0)	15 (0.1)	5 (0.0)	0 (0.0)	0 (0.0)	0 (0.0)	0 (0.0)	0 (0.0)	0 (0.0)	0 (0.0)
**SHARE, 2004** (26329, 97.7)	615 (2.3)	133 (0.5)	133 (0.5)	0 (0.0)	0 (0.0)	10 (0.0)	0 (0.0)	135 (0.5)	135 (0.5)	135 (0.5)	135 (0.5)	135 (0.5)	135 (0.5)	135 (0.5)
**ELSA, 2004** (8671, 78.2)	1894 (21.8)	3 (0.0)	3 (0.0)	0 (0.0)	0 (0.0)	1 (0.0)	789 (9.1)	1 (0.0)	1 (0.0)	1 (0.0)	1 (0.0)	1 (0.0)	1 (0.0)	1 (0.0)
**KLoSA, 2006** (8465, 97.0)	258 (3.0)	0 (0.0)	0 (0.0)	0 (0.0)	0 (0.0)	0 (0.0)	2 (0.0)	1 (0.0)	1 (0.0)	0 (0.0)	0 (0.0)	0 (0.0)	0 (0.0)	0 (0.0)
**MHAS, 2003** (12133, 66.9)	4019 (33.1)	1166 (9.6)	1159 (9.6)	4 (0.0)	0 (0.0)	1 (0.0)	84 (0.7)	50 (0.4)	16 (0.1)	33 (0.3)	9 (0.7)	34 (0.3)	33 (0.3)	20 (0.2)
**LASI**	No data	No data	No data	No data	No data	No data	No data	No data	No data	No data	No data	No data	No data	No data
**CHARLS**	No data	No data	No data	No data	No data	No data	No data	No data	No data	No data	No data	No data	No data	No data
**2015-2018 cross-sectional analysis**														
**HRS, 2018** (16069, 95.0)	235 (1.5)	31 (0.2)	27 (0.2)	804 (5.0)	0 (0.0)	26 (0.2)	3 (0.0)	0 (0.0)	0 (0.0)	0 (0.0)	0 (0.0)	0 (0.0)	0 (0.0)	0 (0.0)
**SHARE, 2017** (38121, 96.8)	1217 (3.2)	419 (1.1)	419 (1.1)	0 (0.0)	0 (0.0)	58 (0.1)	0 (0.0)	170 (0.4)	148 (0.4)	141 (0.4)	184 (0.5)	184 (0.5)	173 (0.4)	168 (0.4)
**ELSA, 2018** (7040, 63.0)	2605 (37.0)	4 (0.0)	4 (0.0)	0 (0.0)	0 (0.0)	1 (0.0)	763 (10.8)	1 (0.0)	0 (0.0)	1 (0.0)	1 (0.0)	0 (0.0)	0 (0.0)	1 (0.0)
**KLoSA, 2018** (6940, 98.5)	108 (1.5)	0 (0.0)	0 (0.0)	0 (0.0)	0 (0.0)	0 (0.0)	0 (0.0)	0 (0.0)	0 (0.0)	0 (0.0)	1 (0.0)	0 (0.0)	0 (0.0)	0 (0.0)
**MHAS, 2018** (15858, 88.7)	1799 (11.3)	1312 (8.3)	1307 (8.2)	20 (0.1)	0 (0.0)	0 (0.0)	234 (1.5)	23 (0.1)	16 (0.1)	14 (0.1)	7 (0.0)	13 (0.1)	11 (0.1)	6 (0.0)
**LASI, 2017** (52393, 89.9)	5307 (10.1)	216 (0.4)	216 (0.4)	0 (0.0)	0 (0.0)	3 (0.0)	0 (0.0)	142 (0.3)	181 (0.3)	140 (0.3)	138 (0.3)	138 (0.3)	138 (0.3)	137 (0.3)
**CHARLS, 2015** (16435, 78.5)	3535 (21.5)	132 (0.8)	160 (1.0)	91 (0.5)	4 (0.0)	0 (0.0)	12 (0.1)	1708 (10.4)	1551 (9.4)	1583 (9.6)	1537 (9.3)	1578 (9.6)	1557 (9.5)	1625 (9.9)

*Results in the darker shade are for surveys from Middle Income Countries

HRS: Health and Retirement Study; SHARE: Survey of Health, Ageing and Retirement in Europe; ELSA: English Longitudinal Study of Ageing; KLoSA: Korean Longitudinal Study of Ageing; MHAS: Mexican Health and Aging Study; LASI: Longitudinal Ageing Study in India; CHARLS: China Health and Retirement Longitudinal Study; BMI: body mass index; ADL: activities of daily living; IADL: instrumental activities of daily living.

[a]Denotes limitations in IADL/ADL

Table S2.* Mean BMI across BMI categories in men and women at the 2002-2006 and 2015-2018 waves of data collection.[a]

	2002-2006				2015-2018			
	Underweight M (SE)	Normal weight M (SE)	Overweight M (SE)	Obesity M (SE)	Underweight M (SE)	Normal weight M (SE)	Overweight M (SE)	Obesity M (SE)
**MEN**								
HRS	17.0 (0.22)	22.9 (0.04)	27.2 (0.03)	33.7 (0.11)	17.0 (0.22)	22.9 (0.05)	27.3 (0.04)	34.8 (0.13)
SHARE	17.3 (0.14)	23.2 (0.03)	27.1 (0.03)	33.0 (0.12)	17.1 (0.21)	23.2 (0.04)	27.2 (0.03)	33.2 (0.11)
ELSA	17.6 (0.13)	23.0 (0.06)	27.3 (0.04)	33.4 (0.12)	16.9 (0.37)	22.9 (0.09)	27.4 (0.06)	34.2 (0.26)
KLoSA	17.3 (0.10)	22.4 (0.03)	26.4 (0.05)	31.3 (0.22)	17.4 (0.13)	22.7 (0.04)	26.5 (0.06)	31.3 (0.27)
MHAS	17.1 (0.28)	22.8 (0.07)	27.2 (0.07)	34.0 (0.36)	16.8 (0.26)	23.0 (0.087)	27.3 (0.05)	33.5 (0.25)
LASI	NA	NA	NA	NA	16.8 (0.02)	21.5 (0.02)	26.9 (0.03)	32.6 (0.12)
CHARLS	NA	NA	NA	NA	17.3 (0.19)	21.9 (0.04)	26.9 (0.05)	32.8 (0.38)
**WOMEN**								
HRS	17.1 (0.07)	22.3 (0.03)	27.2 (0.03)	34.9 (0.12)	17.0 (0.14)	22.4 (0.05)	27.3 (0.04)	35.7 (0.12)
SHARE	17.4 (0.08)	22.5 (0.03)	27.1 (0.03)	33.7 (0.13)	17.3 (0.08)	22.4 (0.03)	27.2 (0.03)	34.0 (0.10)
ELSA	17.4 (0.11)	22.7 (0.05)	27.3 (0.04)	34.2 (0.11)	17.4 (0.12)	22.7 (0.07)	27.3 (0.05)	34.8 (0.19)
KLoSA	17.3 (0.08)	22.3 (0.03)	26.7 (0.04)	31.6 (0.15)	17.4 (0.11)	22.4 (0.04)	26.7 (0.05)	31.9 (0.36)
MHAS	17.1 (0.18)	22.7 (0.09)	27.3 (0.06)	34.2 (0.24)	16.8 (0.30)	22.9 (0.07)	27.5 (0.05)	34.5 (0.17)
LASI	NA	NA	NA	NA	16.6 (0.02)	21.7 (0.02)	27.1 (0.02)	33.4 (0.09)
CHARLS	NA	NA	NA	NA	17.1 (0.27)	22.2 (0.04)	27.0 (0.04)	34.5 (2.26)

*Results in the darker shade are for surveys from Middle Income Countries

SE: Standard error; HRS: Health and Retirement Study; SHARE: Survey of Health, Ageing and Retirement in Europe; ELSA: English Longitudinal Study of Ageing; KLoSA: Korean Longitudinal Study of Ageing; MHAS: Mexican Health and Aging Study; LASI: Longitudinal Ageing Study in India; CHARLS: China Health and Retirement Longitudinal Study; BMI: body mass index.

[a] From analyses weighted to reflect national representativeness.

Table S3.* Characteristics of men and women from the 2002-2006 wave, after imputation for missing data and weighting to obtain national representative data.

			Marital status			Education			Comorbidities						
	BMI Mean	Age Mean	Single	Married cohabiting	Divorced separated widowed	Low	Interm ediate	High	Diabetes	Arthritis	Hyper-tension	Stroke	Cancer	Lung disease	Heart disease
**HRS**															
Men, 44.3%	27.6	66.7	3.0	78.9	18.1	21.6	52.3	26.1	17.4	46.6	47.4	8.0	12.6	7.9	25.4
Women, 55.7%	27.0	68.4	3.4	52.9	43.7	23.1	60.8	16.1	14.1	60.6	49.9	7.3	12.6	7.8	19.1
p-value	<0.001	<0.001		<0.001			<0.001		<0.001	<0.001	0.01	0.16	0.97	0.11	<0.001
**SHARE**															
Men, 45.3%	26.7	64.1	7.7	79.3	13.0	44.2	34.8	21.0	11.5	15.9	30.6	4.1	4.6	6.2	14.2
Women, 54.7%	26.1	66.3	6.7	55.9	37.4	57.9	28.9	13.2	11.0	28.3	36.0	3.2	5.8	5.1	10.4
p-value	<0.001	<0.001		<0.001			<0.001		0.37	<0.001	<0.001	0.02	<0.01	0.01	<0.001
**ELSA**															
Men, 46.5%	27.9	65.6	5.9	78.4	15.7	38.9	44.8	16.3	9.8	27.4	41.1	5.2	6.3	7.1	19.6
Women, 53.5%	28.0	67.1	4.3	60.4	35.3	54.7	36.1	9.2	7.0	41.9	42.1	4.4	8.1	6.0	17.2
p-value	0.41	<0.001		<0.001			<0.001		<0.001	<0.001	0.37	0.08	<0.01	0.05	<0.01
**KLoSA**															
Men, 45.8%	23.2	61.4	0.9	92.6	6.5	48.7	36.4	14.9	13.1	6.5	25.4	4.5	2.3	2.7	4.5
Women, 54.2%	23.3	63.6	0.5	66.7	32.8	79.2	17.3	3.5	12.4	26.2	32.0	3.1	2.6	2.0	5.4
p-value	0.01	<0.001		<0.001			<0.001		0.40	<0.001	<0.001	<0.001	0.41	0.04	0.05
**MHAS**															
Men, 45.9%	26.6	65.2	3.5	80.3	16.2	89.9	2.7	7.4	15.7	22.7	35.5	2.8	1.6	7.3	4.4
Women, 54.1%	27.2	64.6	5.0	54.6	40.4	94.9	0.7	4.4	21.1	33.1	55.3	3.2	2.6	9.4	4.4
p-value	<0.001	0.22		<0.001			<0.001		<0.001	<0.001	<0.001	0.04	0.19	0.03	0.92

*Results in the darker shade are for surveys from Middle Income Countries

HRS: Health and Retirement Study; SHARE: Survey of Health, Ageing and Retirement in Europe; ELSA: English Longitudinal Study of Ageing; KLoSA: Korean Longitudinal Study of Ageing; MHAS: Mexican Health and Aging Study; BMI: body mass index.

Data shown are percentages unless otherwise indicated.

Table S4.* Characteristics of men and women from the 2015-2018 wave, after imputation for missing data and weighting to obtain national representative data.

	BMI Mean	Age Mean	Marital status: Single	Marital status: Married cohabiting	Marital status: Divorced separated widowed	Education: Low	Education: Inter-mediate	Education: High	Comorbidities: Diabetes	Comorbidities: Arthritis	Comorbidities: Hyper-tension	Comorbidities: Stroke	Comorbidities: Cancer	Comorbidities: Lung disease	Comorbidities: Heart disease
**HRS**															
Men, 46.0%	29.0	65.4	7.8	73.3	18.9	10.7	54.8	34.5	26.7	50.7	59.5	7.7	14.6	9.3	26.6
Women, 54.0%	28.8	67.1	7.3	57.2	35.5	11.5	59.6	28.9	23.9	62.7	55.3	7.2	15.4	11.6	20.8
p-value	0.11	<0.001		<0.001			<0.001		<0.001	<0.001	<0.001	0.30	0.27	<0.001	<0.001
**SHARE**															
Men, 46.3%	27.1	66.0	7.9	76.6	15.5	35.1	39.7	25.2	18.5	34.2	52.7	7.3	10.1	11.6	20.8
Women, 53.7%	26.3	67.6	6.2	59.3	34.5	44.0	36.1	19.9	15.6	53.4	51.2	6.5	11.4	10.8	15.6
p-value	<0.001	<0.001		<0.001			<0.001		<0.001	<0.001	0.11	0.05	0.01	0.14	<0.001
**ELSA**															
Men, 47.9%	28.2	64.6	9.2	75.6	15.2	21.9	51.9	26.2	14.4	27.5	41.9	4.7	10.4	6.8	21.9
Women, 52.1%	28.1	65.4	6.4	63.9	29.7	29.8	51.8	18.4	11.2	42.0	37.4	4.2	12.8	5.8	20.4
p-value	0.42	<0.01		<0.001			<0.001		<0.001	<0.001	<0.001	0.34	0.01	0.12	0.16
**KLoSA**															
Men, 46.2%	23.5	65.0	2.0	88.8	9.2	30.3	45.2	24.5	19.2	7.9	37.0	6.1	5.2	2.5	7.5
Women, 53.8%	23.5	66.9	0.7	69.0	30.3	56.6	36.7	6.7	17.4	30.6	41.1	4.7	6.9	2.6	8.2
p-value	0.87	<0.001		<0.001			<0.001		0.10	<0.001	<0.01	0.03	0.01	0.79	0.32
**MHAS**															
Men, 43.5%	27.1	63.8	5.4	80.1	14.5	79.2	4.6	16.2	23.2	15.2	45.4	4.3	2.0	8.6	9.1
Women, 56.5%	28.2	62.6	6.4	61.0	32.6	87.3	3.0	9.7	28.3	28.0	59.0	3.7	4.6	11.5	10.3
p-value	<0.001	<0.001		<0.001			<0.001		<0.001	<0.001	<0.001	0.38	<0.001	<0.001	0.13
**LASI**															
Men, 50.2%	21.8	61.8	1.3	87.6	11.1	66.0	26.9	7.1	13.4	10.9	24.0	2.7	0.5	2.8	4.5
Women, 49.8%	22.9	62.2	0.7	59.4	39.9	87.6	10.2	2.2	12.8	16.3	33.0	1.6	0.8	2.1	3.3
p-value	<0.001	<0.001		<0.001			<0.001		0.14	<0.001	<0.001	<0.001	<0.001	<0.001	<0.001
**CHARLS**															
Men, 49.0%	23.5	63.0	1.4	89.5	9.1	80.0	16.0	4.0	10.6	36.3	33.9	6.2	2.1	16.8	16.1
Women, 51.0%	24.1	63.4	0.2	78.0	21.8	89.9	8.4	1.7	12.4	46.26	37.1	4.9	3.2	13.2	22.4
p-value	0.01	0.15		<0.001			<0.001		0.09	<0.001	0.01	0.16	0.35	<0.001	<0.001

*Results in the darker shade are for surveys from Middle Income Countries

HRS: Health and Retirement Study; SHARE: Survey of Health, Ageing and Retirement in Europe; ELSA: English Longitudinal Study of Ageing; KLoSA: Korean Longitudinal Study of Ageing; MHAS: Mexican Health and Aging Study; BMI: body mass index. Data shown are percentages unless otherwise indicated.

Table S5.* Prevalence of IADL limitations, weighted to be nationally representative.

	Weighted prevalence of IADL limitations[a] (%)							
	Men				Women			
	Underweight	Normal weight	Overweight	Obesity	Underweight	Normal weight	Overweight	Obesity
**2002-2006 (cross-sectional analysis)**								
HRS, 2002	36.7	14.1	10.3	10.0	36.5	13.5	11.7	18.6
SHARE, 2004	51.9	8.7	5.9	6.5	19.5	10.1	11.8	15.1
ELSA, 2004	19.2	11.6	9.5	11.6	17.9	13.6	14.3	18.8
KLoSA, 2006	27.4	14.2	12.4	7.1	29.4	10.4	7.7	9.1
MHAS, 2003	18.2	8.3	6.1	6.5	35.5	13.5	9.7	11.0
LASI	*NA*	*NA*	*NA*	*NA*	*NA*	*NA*	*NA*	*NA*
CHARLS	*NA*	*NA*	*NA*	*NA*	*NA*	*NA*	*NA*	*NA*
**2015-2018 (cross-sectional analysis)**								
HRS, 2018	40.4	14.2	8.9	11.9	37.6	13.3	13.4	16.2
SHARE, 2017	39.3	8.0	6.12	7.6	28.1	10.2	11.0	16.4
ELSA, 2018	40.6	9.8	7.9	10.8	36.5	8.5	9.9	18.3
KLoSA, 2018	22.2	8.7	6.4	8.3	18.1	5.9	4.4	11.2
MHAS, 2018	16.9	8.6	6.1	5.0	18.1	13.7	9.2	11.1
LASI, 2017	35.0	24.2	19.0	18.7	51.7	43.2	36.9	33.2
CHARLS, 2015	29.0	18.9	16.0	10.7	46.3	31.7	27.8	29.6

*Results in the darker shade are for surveys from Middle Income Countries

HRS: Health and Retirement Study; SHARE: Survey of Health, Ageing and Retirement in Europe; ELSA: English Longitudinal Study of Ageing; KLoSA: Korean Longitudinal Study of Ageing; MHAS: Mexican Health and Aging Study; LASI: Longitudinal Ageing Study in India; CHARLS: China Health and Retirement Longitudinal Study

[a]Limitations defined as reporting one or more limitations out of five items (4 items in the MHAS survey) on the IADL scale.

Table S6.* The odds ratio of IADL limitations[a] in underweight, overweight, and obesity groups compared to normal weight men and women in cross-sectional analyses using data from 2002-2006 and 2015-2018.

	BMI CATEGORIES														
	**Underweight**					**Overweight**					**Obesity**				
	**OR (95% CI)[b] 2002-2006**	**OR (95% CI)[b] 2015-2018**	**p[c]**	**I^2 [d]**	**I^2 [e]**	**OR (95% CI)[b] 2002-2006**	**OR (95% CI)[b] 2015-2018**	**p[c]**	**I^2 [d]**	**I^2 [e]**	**OR (95% CI)[b] 2002-2006**	**OR (95% CI)[b] 2015-2018**	**p[c]**	**I^2 [d]**	**I^2 [e]**
**MEN**															
HRS	**2.39 (1.23, 4.65)**	**3.04 (1.40, 6.60)**	0.65			0.83 (0.68, 1.02)	**0.63 (0.49, 0.83)**	0.12			**0.74 (0.58, 0.96)**	**0.69 (0.52, 0.93)**	0.73		
SHARE	**4.20 (1.61, 10.99)**	**5.01 (1.64, 15.30)**	0.86			**0.75 (0.57, 0.97)**	0.81 (0.62, 1.06)	0.68			0.72 (0.50, 1.04)	1.04 (0.75, 1.44)	0.17		
ELSA	1.31 (0.32, 5.30)	**4.09 (1.05, 15.95)**	0.25			0.84 (0.60, 1.18)	0.80 (0.50, 1.27)	0.86			0.87 (0.60, 1.25)	0.74 (0.44, 1.23)	0.61		
KLoSA	1.42 (0.92, 2.18)	1.73 (0.76, 3.94)	0.68			0.97 (0.73, 1.30)	0.82 (0.54, 1.26)	0.51			0.49 (0.10, 2.39)	0.55 (0.17, 1.81)	0.91		
**ALL HIC**	**2.00 (1.22, 3.29)**	**2.86 (1.78, 4.59)**		42.1	0.0	**0.84 (0.74, 0.95)**	**0.74 (0.63, 0.87)**		0.0	0.0	**0.76 (0.63, 0.91)**	0.80 (0.62, 1.05)		0.0	33.3
MHAS	1.53 (0.33, 7.17)	1.36 (0.52, 3.55)	0.90			0.90 (0.50, 1.63)	0.79 (0.51, 1.20)	0.72			0.94 (0.44, 1.98)	0.67 (0.38, 1.18)	0.48		
LASI	NA	**1.38 (1.25, 1.53)**				NA	**0.86 (0.76, 0.97)**				NA	0.93 (0.72, 1.19)			
CHARLS	NA	1.27 (0.90, 1.78)				NA	0.89 (0.69, 1.16)				NA	0.56 (0.32, 1.00)			
**ALL MIC**		**1.37 (1.25, 1.51)**			0.0		**0.86 (0.77, 0.96)**			0.0		0.77 (0.55, 1.06)			39.6
**ALL**	**1.94 (1.25, 3.01)**	**1.78 (1.26, 2.52)**		30.4	62.6	**0.84 (0.74, 0.95)**	**0.82 (0.74, 0.90)**		0.0	5.4	**0.77 (0.65, 0.92)**	**0.80 (0.67, 0.96)**		0.0	25.3
***p*-value[f]**		**<0.01**					0.13					0.83			
**WOMEN**															
HRS	**2.51 (1.79, 3.52)**	**2.90 (1.87, 4.48)**	0.61			0.87 (0.73, 1.05)	0.98 (0.79, 1.21)	0.43			**1.49 (1.23, 1.81)**	1.12 (0.91, 1.39)	0.05		
SHARE	1.41 (0.78, 2.55)	**2.36 (1.48, 3.76)**	0.16			**1.29 (1.03, 1.64)**	1.03 (0.83, 1.28)	0.15			**1.84 (1.40, 2.40)**	**1.61 (1.26, 2.04)**	0.44		
ELSA	0.93 (0.35, 2.48)	**7.18 (3.09, 16.64)**	**<0.01**			0.99 (0.75, 1.29)	1.04 (0.70, 1.54)	0.82			1.29 (0.96, 1.71)	**1.55 (1.08, 2.25)**	0.43		
KLoSA	**1.66 (1.13, 2.46)**	**1.93 (1.08, 3.45)**	0.68			0.93 (0.70, 1.25)	**0.64 (0.44, 0.95)**	0.13			1.28 (0.60, 2.74)	1.76 (0.62, 5.01)	0.63		
**ALL HIC**	**1.75 (1.23, 2.50)**	**2.86 (1.87, 4.38)**		50.2	57.5	1.01 (0.84, 1.21)	0.96 (0.84, 1.09)		57.3	0.0	**1.51 (1.29, 1.77)**	**1.40 (1.11, 1.77)**		20.0	50.4
MHAS	2.07 (0.72, 6.00)	1.54 (0.57, 4.14)	0.69			0.80 (0.49, 1.29)	0.95 (0.65, 1.38)	0.59			0.92 (0.52, 1.63)	1.26 (0.86, 1.86)	0.38		
LASI	NA	**1.25 (1.14, 1.36)**				NA	**0.88 (0.81, 0.96)**				NA	**0.82 (0.72, 0.92)**			
CHARLS	NA	1.28 (0.80, 2.06)				NA	0.85 (0.70, 1.02)				NA	0.91 (0.59, 1.39)			
**ALL MIC**		**1.25 (1.15, 1.36)**			0.0		**0.88 (0.81, 0.95)**			0.0		0.93 (0.72, 1.21)			55.0
**ALL**	**1.79 (1.31, 2.46)**	**2.07 (1.38, 3.10)**		37.9	83.7	0.99 (0.83, 1.17)	**0.90 (0.84, 0.96)**		50.0	0.0	**1.44 (1.20, 1.73)**	1.18 (0.94, 1.49)		35.5	77.4
***p*-value[f]**		**<0.01**					0.27					**0.02**			

*Results in the darker shade are for surveys from Middle Income Countries

IADL: instrumental activities of daily living; HRS: Health and Retirement Study; SHARE: Survey of Health, Ageing and Retirement in Europe; ELSA: English Longitudinal Study of Ageing; KLoSA: Korean Longitudinal Study of Ageing; HIC: high-income countries; MHAS: Mexican Health and Aging Study; LASI: Longitudinal Ageing Study in India; CHARLS: China Health and Retirement Longitudinal Study; MIC: middle-income countries. [a]Limitations defined as reporting one or more limitations out of five items (4 items in the MHAS cohort) on the IADL scale.

[b]Logistic regression model adjusted for age, age^2, marital status, education, and prevalence of diabetes, arthritis, hypertension, stroke, cancer, lung disease, and heart disease. Reference group is normal weight individuals. [c]p-value for difference between estimates from 2002-2006 and 2015-2018 cross-sectional analysis with 2002-2006 being the reference. [d]2002-2006 cross-sectional analysis. [e]2015-2018 cross-sectional analysis. [f]p-value of Q test for difference in meta-analysis estimates between HIC and MIC.

Table S7.* Prevalence of ADL limitations, weighted to be nationally representative.

	Weighted prevalence of ADL limitations[a] (%)							
	Men				Women			
	Underweight	Normal weight	Overweight	Obesity	Underweight	Normal weight	Overweight	Obesity
**2002-2006 (cross-sectional analysis)**								
HRS, 2002	31.6	11.9	9.9	15.4	27.7	13.2	15.2	24.5
SHARE, 2004	44.2	9.2	8.4	13.1	20.5	10.5	11.8	19.4
ELSA, 2004	14.5	14.7	16.2	28.2	19.6	15.6	20.4	31.1
KLoSA, 2006	11.9	4.0	2.8	6.8	17.5	4.8	2.8	4.0
MHAS, 2003	14.0	8.8	6.9	7.9	32.7	10.9	9.7	12.3
LASI	*NA*	*NA*	*NA*	*NA*	*NA*	*NA*	*NA*	*NA*
CHARLS	*NA*	*NA*	*NA*	*NA*	*NA*	*NA*	*NA*	*NA*
**2015-2018 (cross-sectional analysis)**								
HRS, 2018	25.9	11.6	10.0	16.5	31.4	11.6	13.3	20.0
SHARE, 2017	36.8	8.6	8.2	13.9	26.3	10.5	11.9	20.0
ELSA, 2018	43.6	11.3	11.7	20.8	32.8	10.5	13.8	26.7
KLoSA, 2018	12.0	2.4	1.4	10.2	10.9	3.0	2.4	3.8
MHAS, 2018	28.5	11.7	11.1	12.1	20.6	18.1	13.0	18.7
LASI, 2017	17.0	12.9	12.5	13.1	22.0	18.4	18.4	21.1
CHARLS, 2015	20.9	15.5	14.6	13.5	26.7	22.4	24.5	31.0

*Results in the darker shade are for surveys from Middle Income Countries

HRS: Health and Retirement Study; SHARE: Survey of Health, Ageing and Retirement in Europe; ELSA: English Longitudinal Study of Ageing; KLoSA: Korean Longitudinal Study of Ageing; MHAS: Mexican Health and Aging Study; LASI: Longitudinal Ageing Study in India; CHARLS: China Health and Retirement Longitudinal Study

[a]Limitations defined as reporting one or more limitations out of 5 items on the ADL scale.

Table S8.* The odds ratio of ADL limitations[a] in underweight, overweight, and obesity groups compared to normal weight men and women in cross-sectional analyses using data from 2002-2006 and 2015-2018.

	BMI CATEGORIES														
	Underweight					Overweight					Obesity				
	OR (95% CI)[b] 2002-2006	OR (95% CI)[b] 2015-2018	p[c]	I^2 [d]	I^2 [e]	OR (95% CI)[b] 2002-2006	OR (95% CI)[b] 2015-2018	p[c]	I^2 [d]	I^2 [e]	OR (95% CI)[b] 2002-2006	OR (95% CI)[b] 2015-2018	p[c]	I^2 [d]	I^2 [e]
**MEN**															
HRS	**2.48 (1.20, 5.11)**	1.77 (0.71, 4.39)	0.57			0.95 (0.77, 1.17)	0.90 (0.69, 1.19)	0.80			**1.47 (1.16, 1.85)**	1.30 (0.98, 1.74)	0.54		
SHARE	2.86 (0.95, 8.62)	**4.03 (1.58, 10.30)**	0.60			1.03 (0.81, 1.30)	0.98 (0.76, 1.24)	0.81			**1.59 (1.18, 2.16)**	**1.72 (1.32, 2.25)**	0.57		
ELSA	0.78 (0.15, 3.94)	**4.80 (1.42, 16.26)**	**0.05**			1.16 (0.88, 1.54)	1.07 (0.70, 1.63)	0.74			**2.11 (1.56, 2.87)**	**1.67 (1.07, 2.61)**	0.38		
KLoSA	1.63 (0.88, 3.02)	2.69 (0.85, 8.54)	0.46			0.84 (0.49, 1.43)	0.66 (0.28, 1.56)	0.65			2.10 (0.31, 14.07)	2.70 (0.82, 8.95)	0.83		
**ALL HIC**	**1.93 (1.27, 2.94)**	**2.95 (1.76, 4.93)**		0.0	0.0	1.01 (0.88, 1.15)	0.95 (0.81, 1.12)		0.0	0.0	**1.68 (1.36, 2.08)**	**1.57 (1.28, 1.91)**		33.5	12.7
MHAS	1.07 (0.25, 4.61)	**2.36 (1.16, 4.79)**	0.33			0.89 (0.52, 1.51)	1.10 (0.77, 1.56)	0.52			0.96 (0.51, 1.83)	1.34 (0.88, 2.05)	0.40		
LASI	NA	**1.22 (1.08, 1.39)**				NA	0.99 (0.86, 1.15)				NA	1.06 (0.80, 1.42)			
CHARLS	NA	1.05 (0.69, 1.59)				NA	1.03 (0.78, 1.35)				NA	0.95 (0.47, 1.90)			
**ALL MIC**		**1.23 (1.09, 1.39)**			0.0		1.01 (0.90, 1.14)			0.0		1.12 (0.90, 1.41)			0.0
**ALL**	**1.85 (1.24, 2.76)**	**1.89 (1.22, 2.91)**		0.0	72.3	1.00 (0.88, 1.14)	0.99 (0.90, 1.09)		0.0	0.0	**1.60 (1.28, 1.99)**	**1.38 (1.14, 1.65)**		38.2	34.4
***p*-value[f]**		**<0.01**					0.55					**0.03**			
**WOMEN**															
HRS	**1.59 (1.12, 2.25)**	**2.58 (1.59, 4.21)**	0.11			**1.22 (1.03, 1.44)**	1.13 (0.91, 1.40)	0.60			**2.10 (1.76, 2.51)**	**1.69 (1.37, 2.09)**	0.13		
SHARE	1.61 (0.91, 2.84)	**2.07 (1.29, 3.34)**	0.46			1.15 (0.93, 1.42)	1.07 (0.88, 1.31)	0.64			**2.27 (1.80, 2.86)**	**1.83 (1.47, 2.29)**	0.21		
ELSA	0.88 (0.37, 2.05)	**4.38 (1.93, 9.94)**	**0.01**			**1.34 (1.07, 1.69)**	1.24 (0.88, 1.75)	0.72			**2.23 (1.74, 2.87)**	**2.14 (1.48, 3.09)**	0.85		
KLoSA	**2.03 (1.27, 3.23)**	**2.15 (1.12, 4.13)**	0.89			0.68 (0.44, 1.06)	0.69 (0.41, 1.15)	0.98			1.11 (0.39, 3.21)	1.03 (0.30, 3.59)	0.93		
**ALL HIC**	**1.62 (1.27, 2.06)**	**2.46 (1.85, 3.26)**		0.0	0.0	1.14 (0.95, 1.38)	1.09 (0.96, 1.24)		58.0	0.0	**2.16 (1.91, 2.44)**	**1.80 (1.56, 2.07)**		0.0	0.0
MHAS	2.30 (0.76, 6.92)	1.08 (0.42, 2.78)	0.33			0.97 (0.58, 1.60)	0.88 (0.65, 1.18)	0.75			1.34 (0.81, 2.22)	**1.41 (1.04, 1.90)**	0.87		
LASI	NA	**1.15 (1.04, 1.28)**				NA	1.06 (0.95, 1.18)				NA	**1.30 (1.12, 1.51)**			
CHARLS	NA	0.94 (0.62, 1.42)				NA	1.14 (0.93, 1.38)				NA	**1.57 (1.09, 2.28)**			
**ALL MIC**		**1.14 (1.03, 1.26)**			0.0		1.06 (0.97, 1.16)			0.0		**1.35 (1.19, 1.53)**			0.0
**ALL**	**1.64 (1.30, 2.08)**	**1.72 (1.16, 2.53)**		0.0	81.2	1.13 (0.97, 1.33)	1.07 (0.99, 1.15)		44.0	0.0	**2.10 (1.87, 2.37)**	**1.59 (1.37, 1.84)**		0.0	48.8
***p*-value[f]**		**<0.01**					0.73					**<0.01**			

*Results in the darker shade are for surveys from Middle Income Countries

ADL: basic activities of daily living; HRS: Health and Retirement Study; SHARE: Survey of Health, Ageing and Retirement in Europe; ELSA: English Longitudinal Study of Ageing; KLoSA: Korean Longitudinal Study of Ageing; HIC: high-income countries; MHAS: Mexican Health and Aging Study; LASI: Longitudinal Ageing Study in India; CHARLS: China Health and Retirement Longitudinal Study; MIC: middle-income countries.

[a]Limitations defined as reporting one or more limitations out of five items on the ADL scale.

[b]Logistic regression model adjusted for age, age^2, marital status, education, and prevalence of diabetes, arthritis, hypertension, stroke, cancer, lung disease, and heart disease. Reference group is normal weight individuals. [c]p-value for difference between estimates from 2002-2006 and 2015-2018 cross-sectional analysis with 2002-2006 being the reference.

[d]2002-2006 cross-sectional analysis. [e]2015-2018 cross-sectional analysis. [f]p-value of Q test difference in meta-analysis estimates between HIC and MIC.

Table S9*. P-values of the test for interaction to examine whether age (<65 years vs ≥65 years) modifies the association between BMI and IADL/ADL limitations in men and women (2015-2018).

	MEN		WOMEN	
	IADL limitations[a]	ADL limitations[b]	IADL limitations[a]	ADL limitations[b]
HRS	0.61	0.20	0.08	0.14
SHARE	0.78	0.33	0.57	0.99
ELSA	0.94	0.74	0.44	0.66
KLoSA	0.03	0.33	0.01	0.66
MHAS	0.22	0.82	0.85	0.15
LASI	0.36	0.81	0.84	0.12
CHARLS	0.68	0.96	0.44	0.84

*Results in the darker shade are for surveys from Middle Income Countries

HRS: Health and Retirement Study; SHARE: Survey of Health, Ageing and Retirement in Europe; ELSA: English Longitudinal Study of Ageing; KLoSA: Korean Longitudinal Study of Ageing; MHAS: Mexican Health and Aging Study; LASI: Longitudinal Ageing Study in India; CHARLS: China Health and Retirement Longitudinal Study.

[a]Limitations defined as reporting one or more limitations out of five items (4 items in the MHAS) on the IADL scale.

[b]Limitations defined as reporting one or more limitations out of five items on the ADL scale.

Figure S1. Flow chart of sample selection

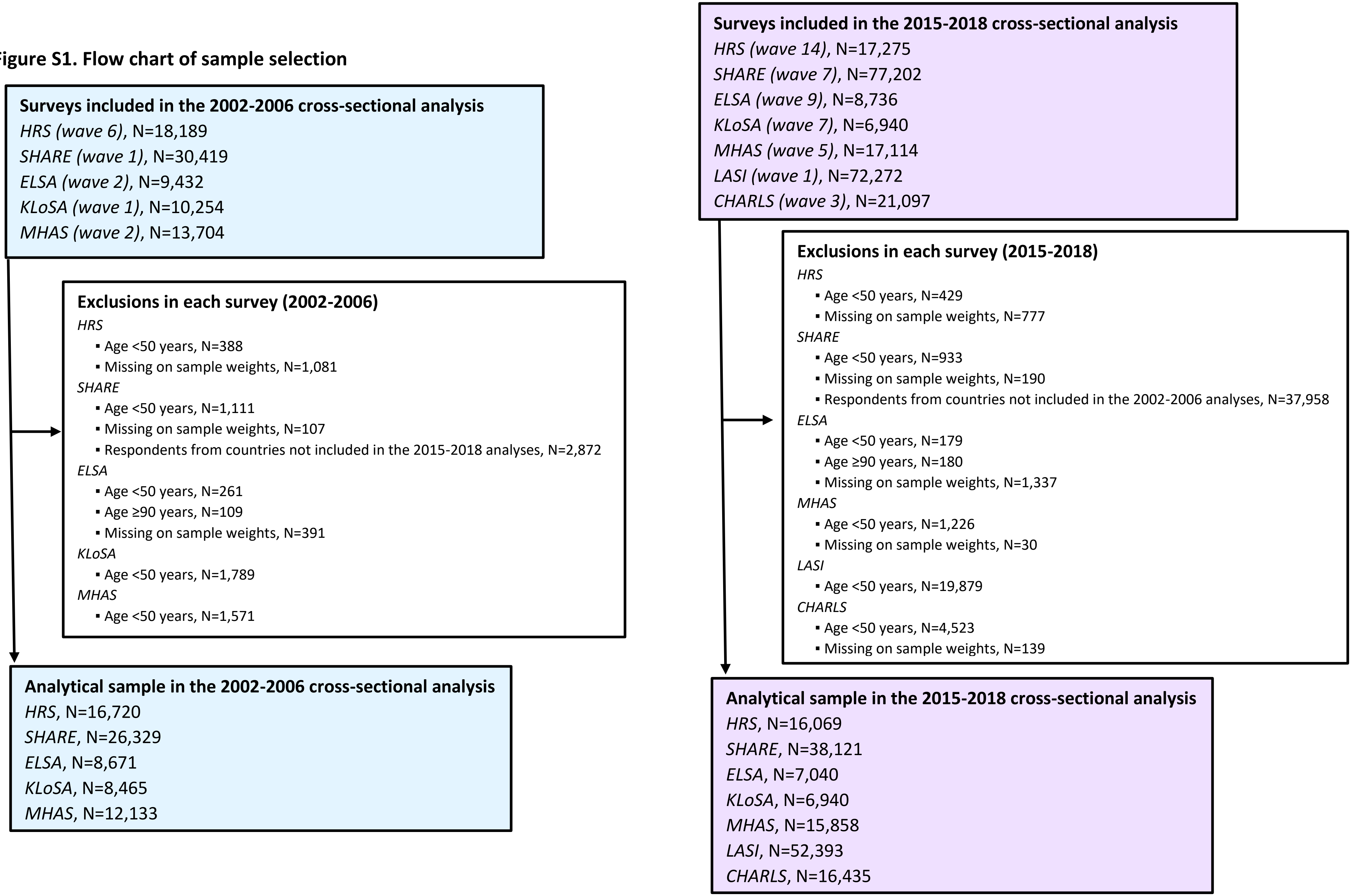

HRS: Health and Retirement Study; SHARE: Survey of Health, Ageing and Retirement in Europe; ELSA: English Longitudinal Study of Ageing; KLoSA: Korean Longitudinal Study of Ageing; MHAS: Mexican Health and Aging Study; LASI: Longitudinal Ageing Study in India; CHARLS: China Health and Retirement Longitudinal Study.

Figure S2*. Association between BMI and individual IADL limitations[a] in men and women using data from 2015-2018.[b]

2.1) Difficulty making telephone calls.

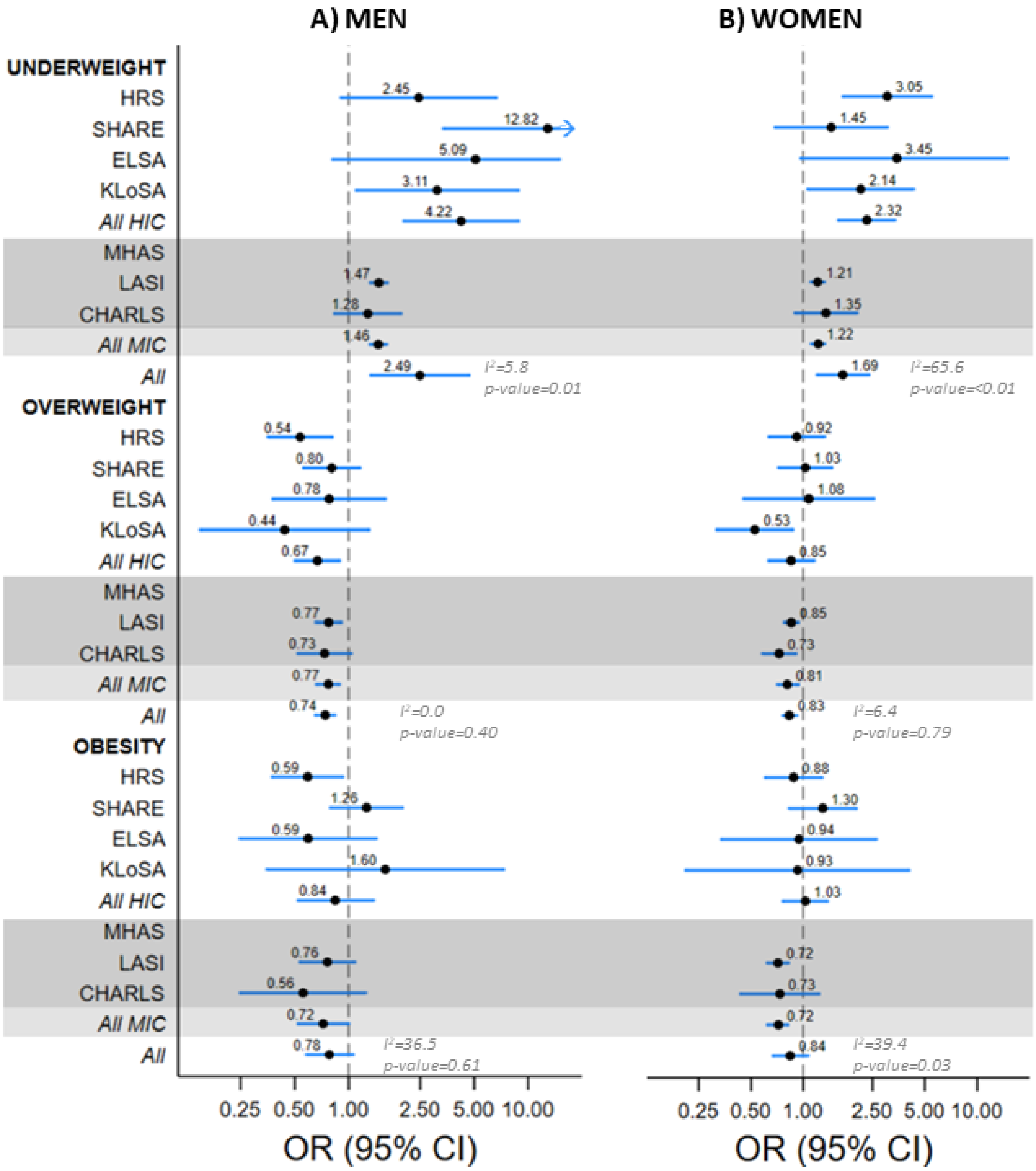

2.2) Difficulty managing money.

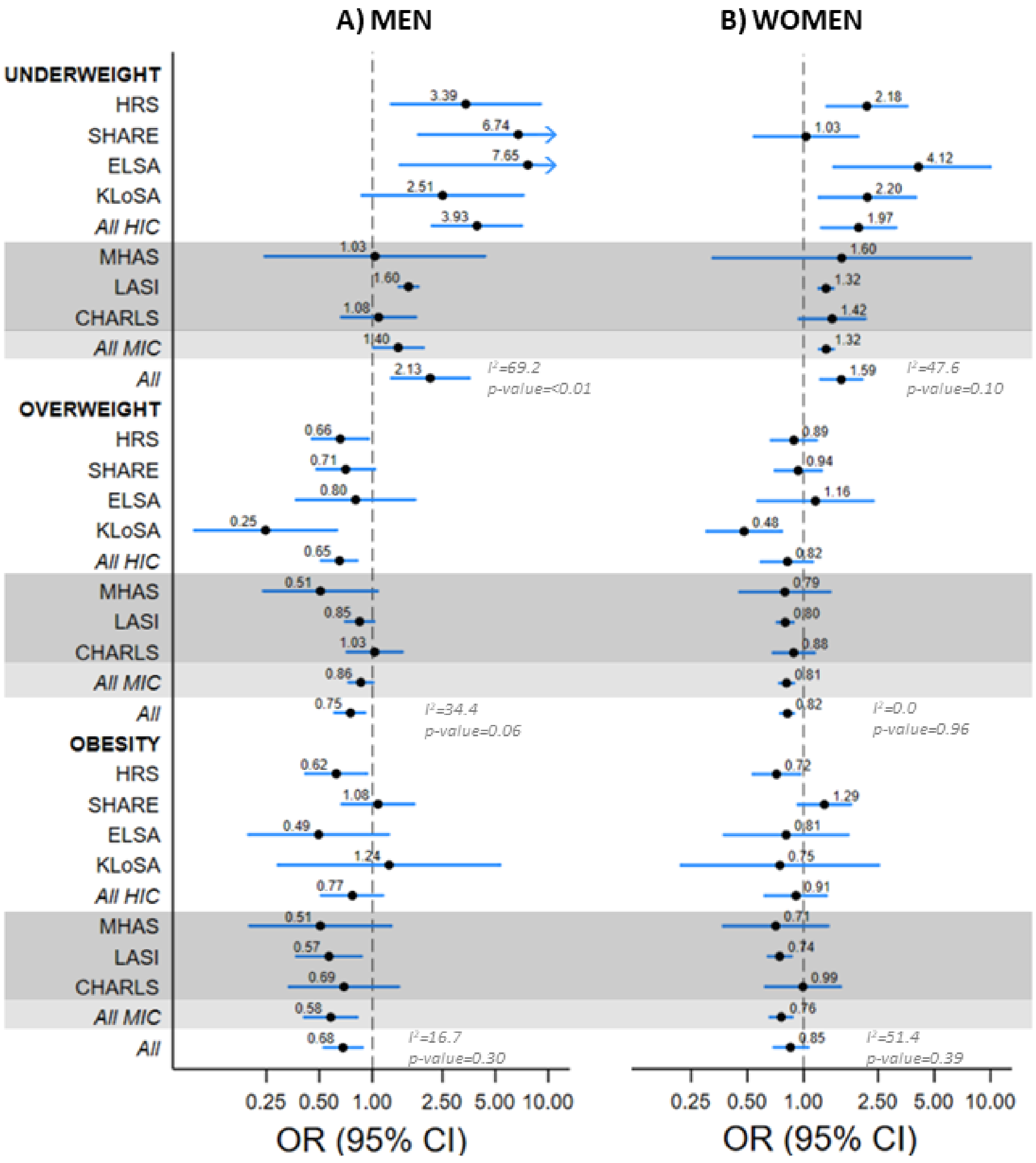

2.3) Difficulty taking medications.

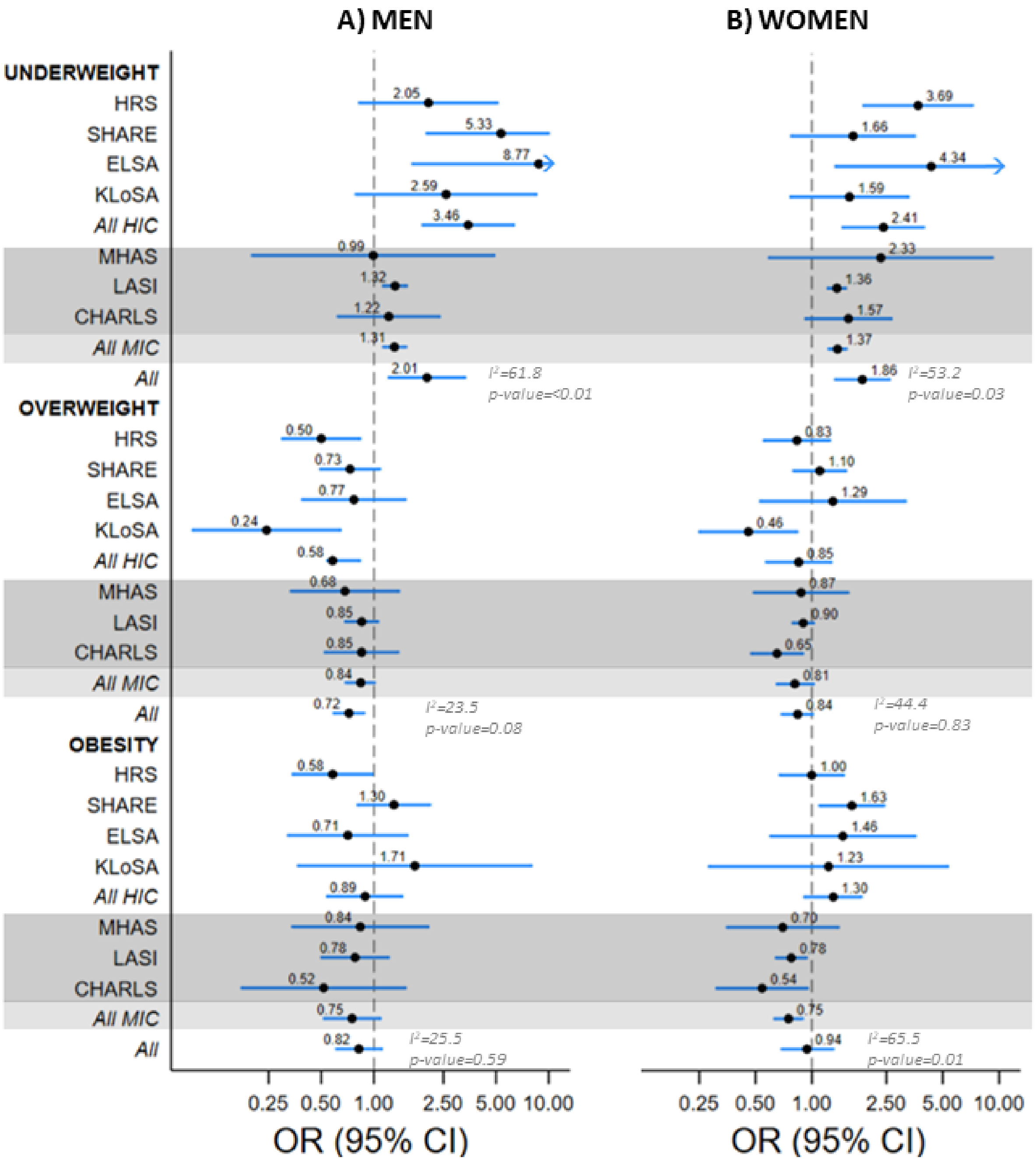

2.4) Difficulty shopping for groceries.

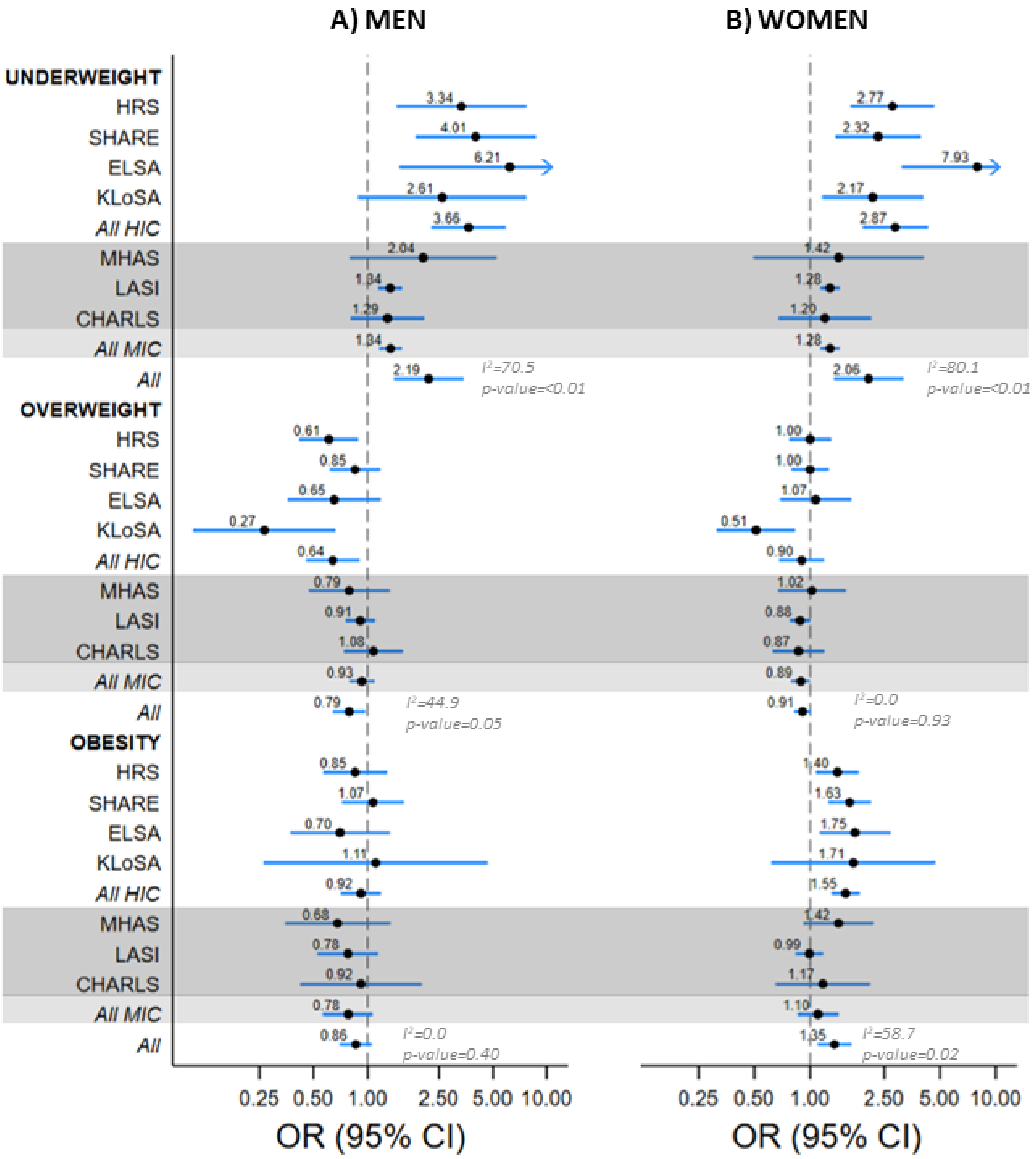

2.5) Difficulty preparing hot meals.

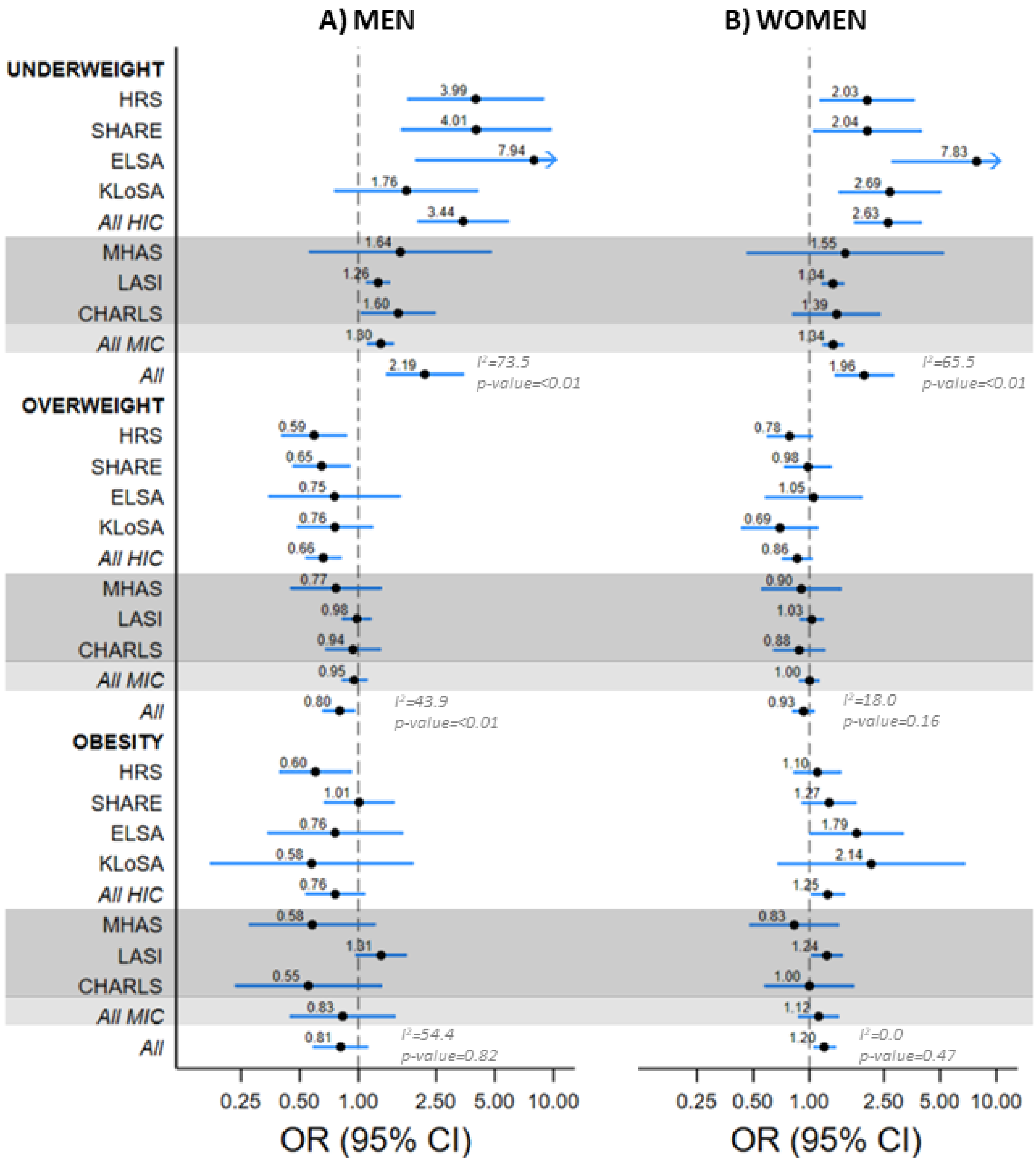

*Results in the darker shade are for surveys from Middle Income Countries
BMI: body mass index. IADL: instrumental activities of daily living; HRS: Health and Retirement Study; SHARE: Survey of Health, Ageing and Retirement in Europe; ELSA: English Longitudinal Study of Ageing; KLoSA: Korean Longitudinal Study of Ageing; MHAS: Mexican Health and Aging Study; LASI: Longitudinal Ageing Study in India; CHARLS: China Health and Retirement Longitudinal Study.

[a]Limitation defined as reporting difficulty in each activity. "Making telephone calls" was not included in MHAS.

[b] Logistic regression model adjusted for age, age^2, marital status, education, and prevalence of diabetes, arthritis, hypertension, stroke, cancer, lung disease, and heart disease.

p-values for differences in meta-analysis estimates between HIC and MIC are provided.

Arrows denote estimates where the upper limit of the confidence interval exceeds the X-axis range.

Figure S3*. Association between BMI and individual ADL limitations[a] in men and women using data from 2015-2018.[b]

3.1) Difficulty bathing.

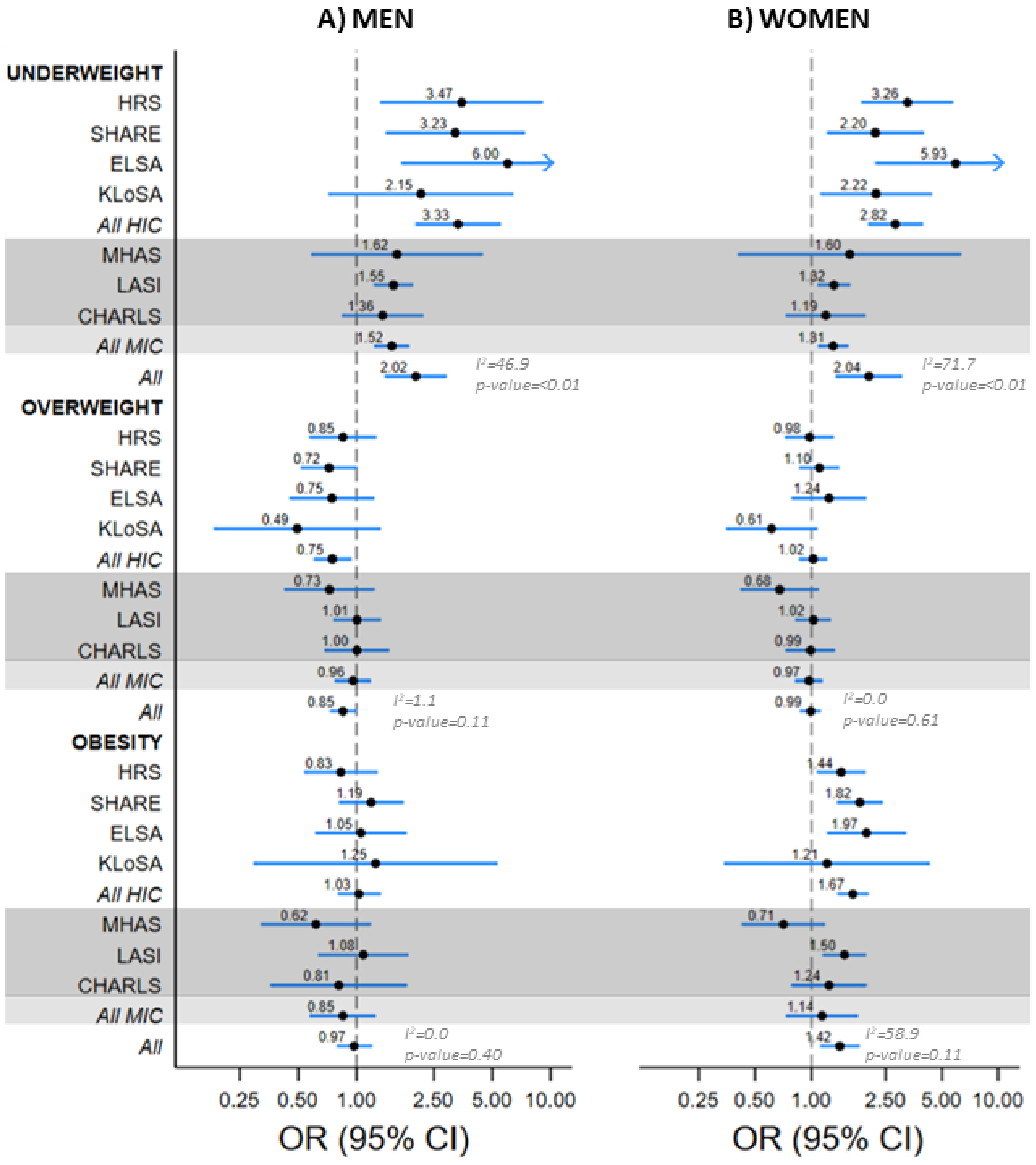

3.2) Difficulty dressing.

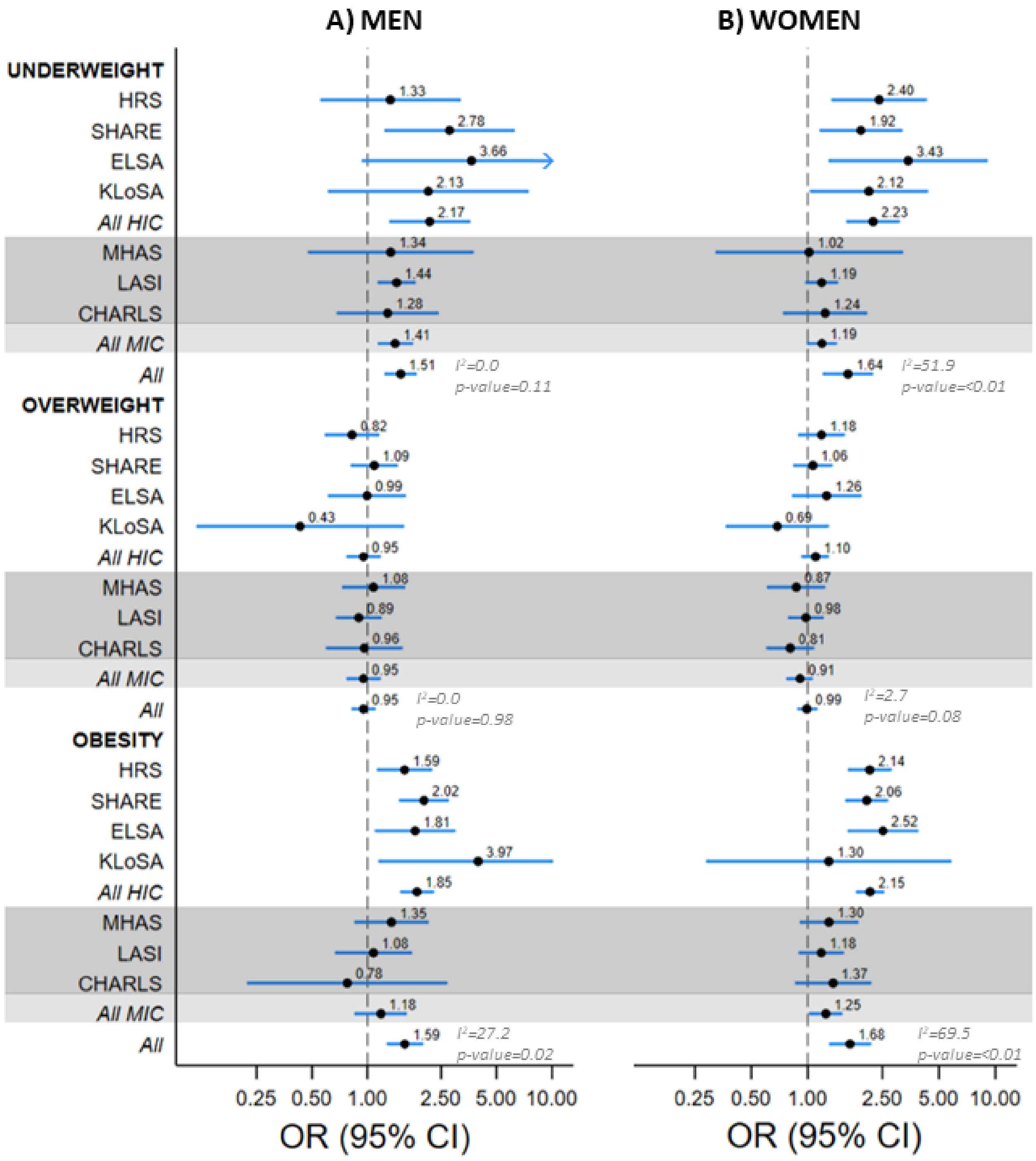

3.3) Difficulty eating.

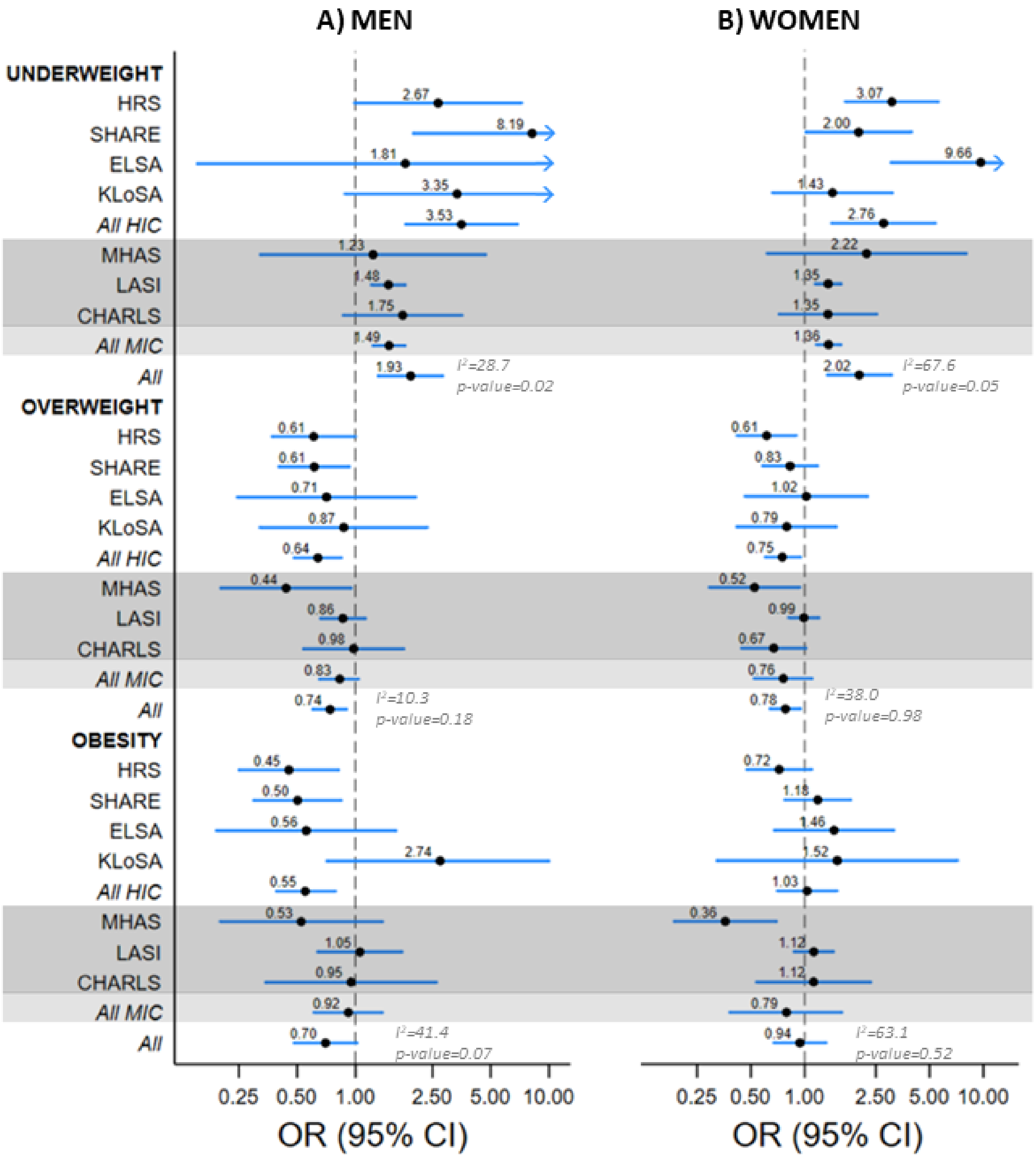

3.4) Difficulty getting in/out of bed.

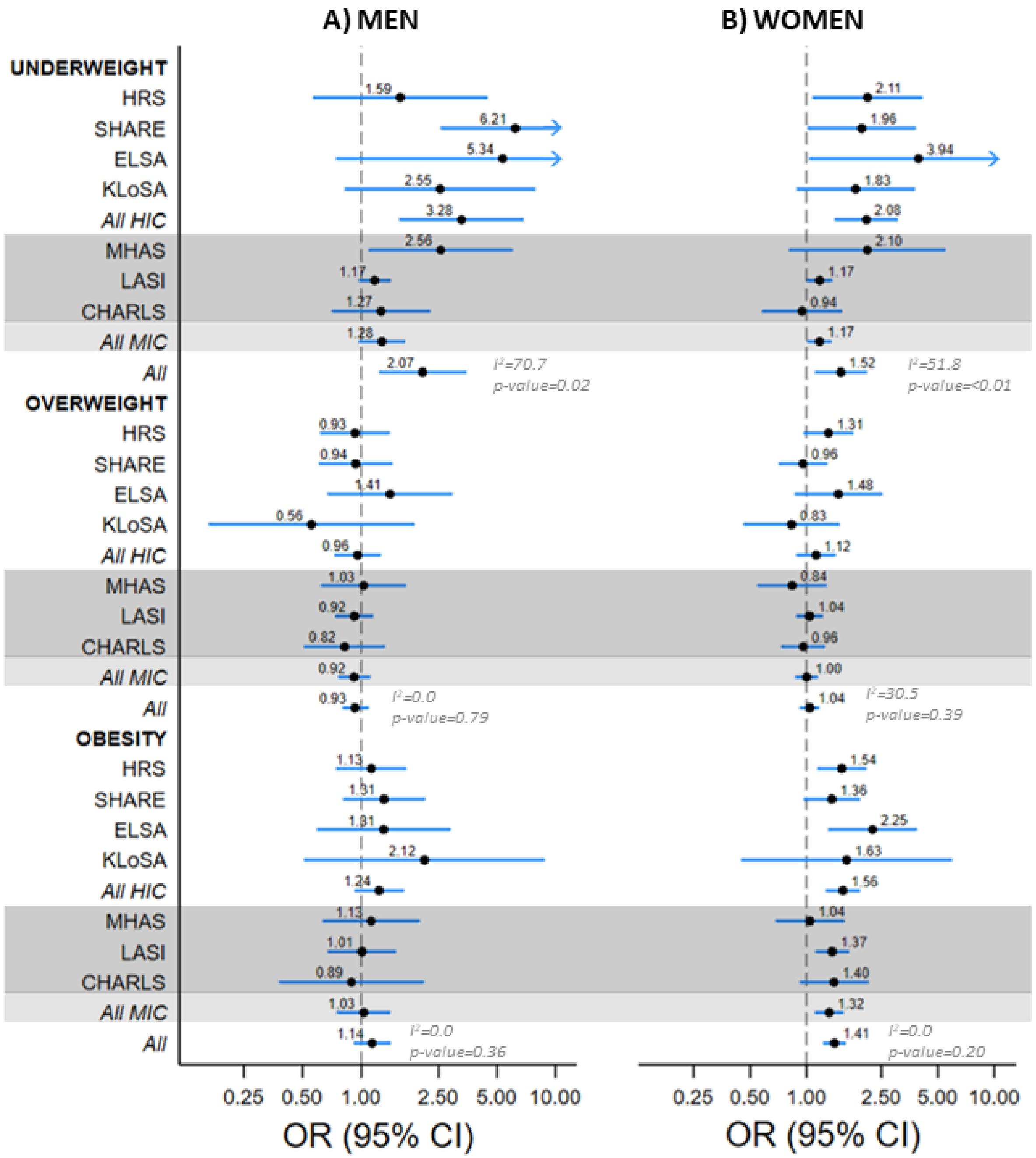

3.5) Difficulty using the toilet.

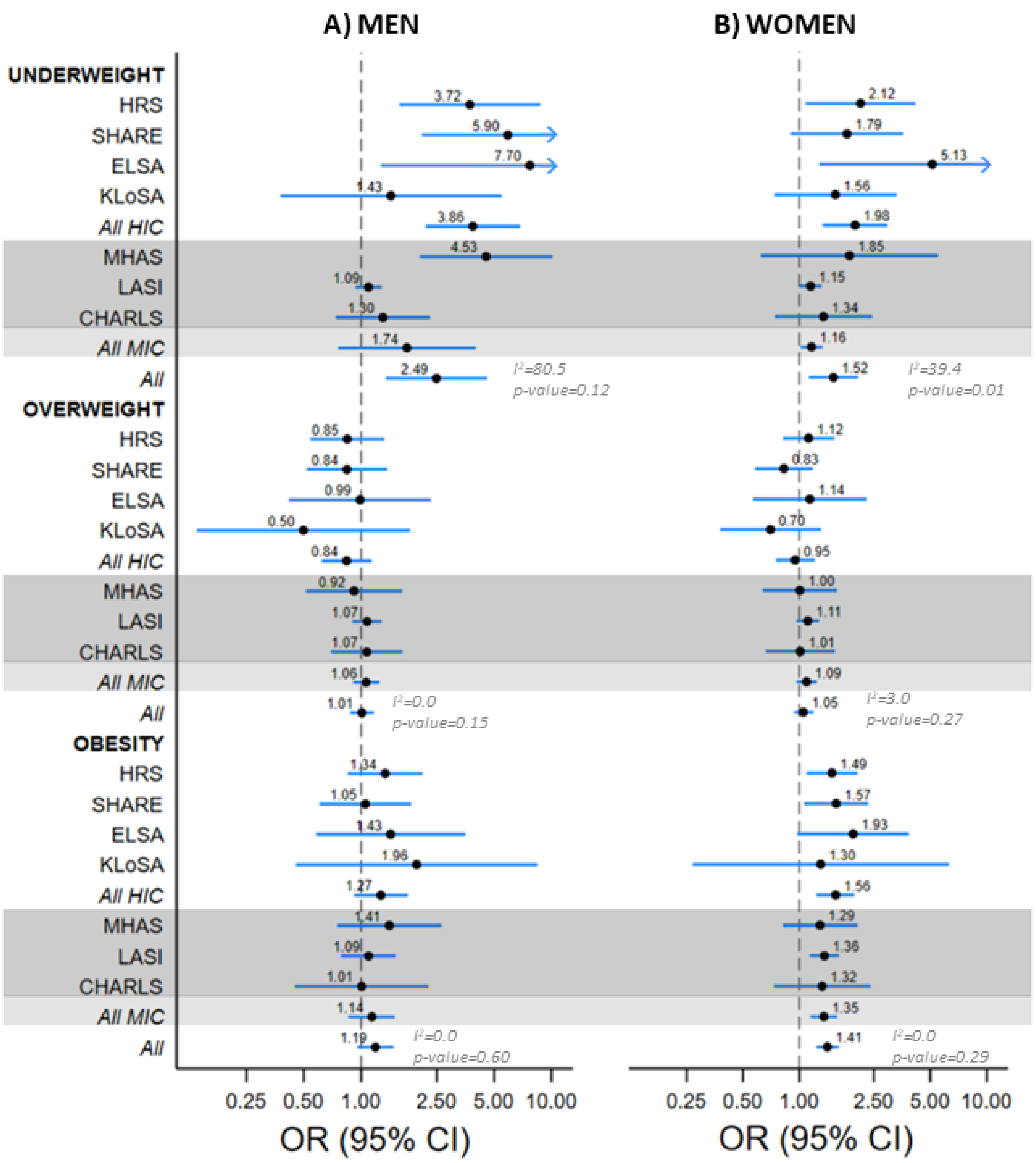

*Results in the darker shade are for surveys from Middle Income Countries

BMI: body mass index. IADL: instrumental activities of daily living; HRS: Health and Retirement Study; SHARE: Survey of Health, Ageing and Retirement in Europe; ELSA: English Longitudinal Study of Ageing; KLoSA: Korean Longitudinal Study of Ageing; MHAS: Mexican Health and Aging Study; LASI: Longitudinal Ageing Study in India; CHARLS: China Health and Retirement Longitudinal Study.

[a]Limitation defined as reporting difficulty in each activity. In CHARLS, “using the toilet” was defined as “controlling urination/defecation”.

[b] Logistic regression model adjusted for age, age^2, marital status, education, and prevalence of diabetes, arthritis, hypertension, stroke, cancer, lung disease, and heart disease.

p-values for differences in meta-analysis estimates between HIC and MIC are provided.

Arrows denote estimates where the upper limit of the confidence interval exceeds the X-axis range.

Figure S4*. Association between BMI (using alternative WHO thresholds for Asia-Pacific countries)[a] and IADL limitations[b] in men and women using data from 2015-2018.[c]

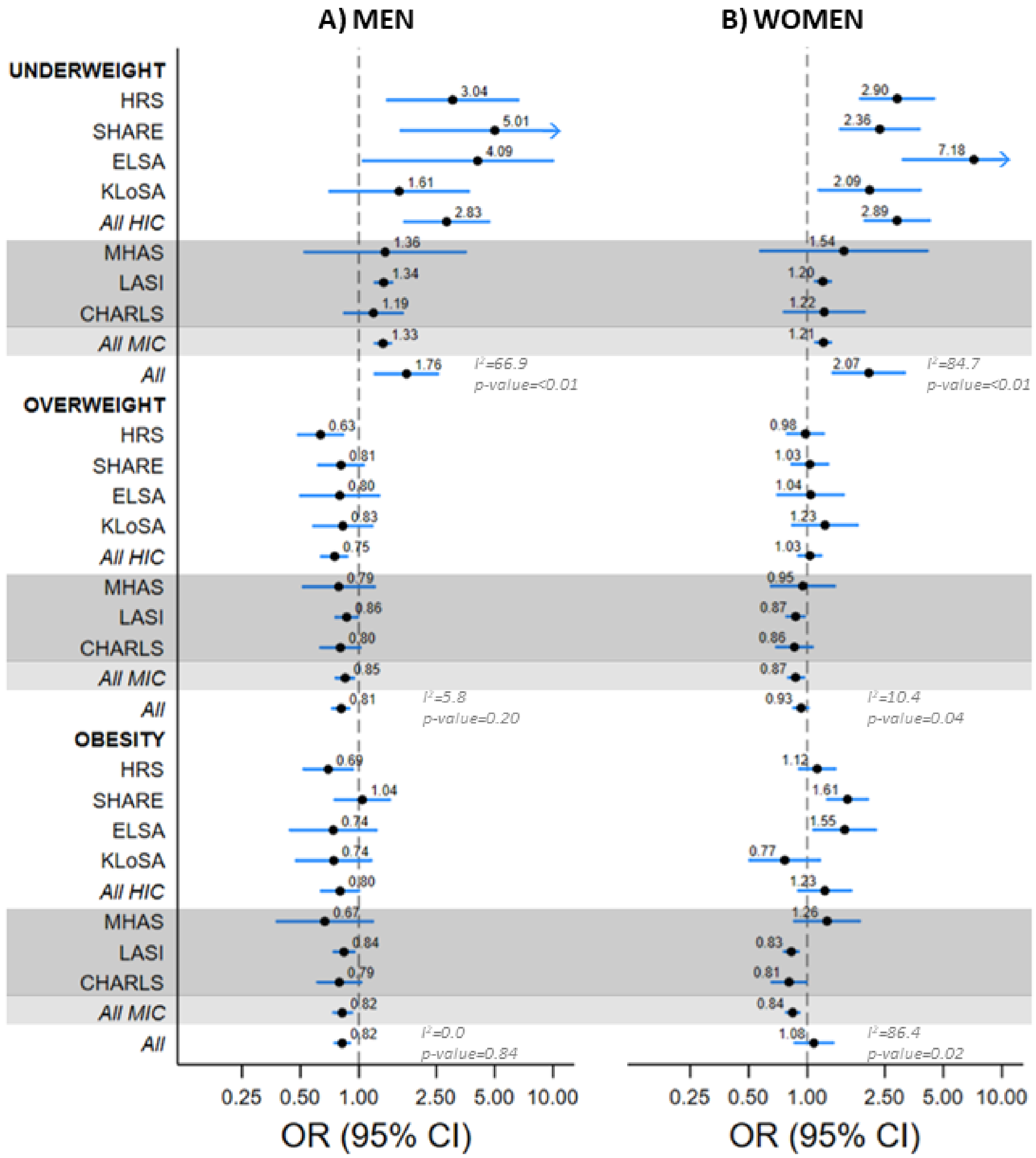

*Results in the darker shade are for surveys from Middle Income Countries

BMI: body mass index; IADL: instrumental activities of daily living; HRS: Health and Retirement Study; SHARE: Survey of Health, Ageing and Retirement in Europe; ELSA: English Longitudinal Study of Ageing; KLoSA: Korean Longitudinal Study of Ageing; MHAS: Mexican Health and Aging Study; LASI: Longitudinal Ageing Study in India; CHARLS: China Health and Retirement Longitudinal Study.

[a]BMI categories were classified as underweight (<18.5 kg/m²), normal weight (≥18.5 kg/m² to <23 kg/m²), overweight (≥23 kg/m² to <25 kg/m²), and obesity (≥25 kg/m²) in the KLoSA, LASI and CHARLS surveys.

[b]Limitations defined as reporting one or more limitations out of five items (4 items in the MHAS) on the IADL scale.

[c] Logistic regression model adjusted for age, age², marital status, education, and prevalence of diabetes, arthritis, hypertension, stroke, cancer, lung disease, and heart disease.

p-values of Q test for difference in meta-analysis estimated between HIC and MIC.

Arrows denote estimates where the upper limit of the confidence interval exceeds the X-axis range.

Figure S5*. Association between BMI (using alternative WHO thresholds for Asia-Pacific countries)[a] and ADL limitations[b] in men and women using data from 2015-2018.[c]

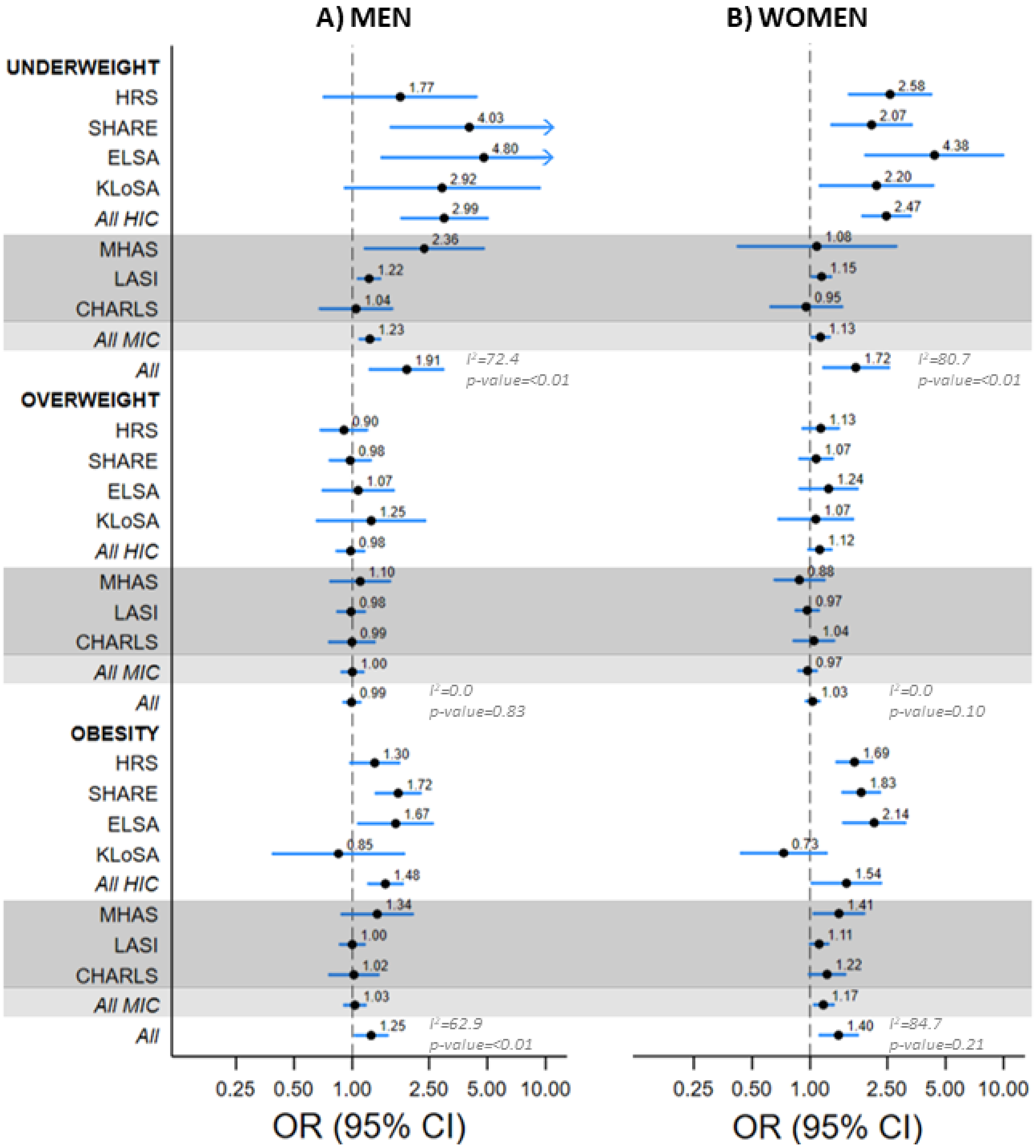

*Results in the darker shade are for surveys from Middle Income Countries

BMI: body mass index; ADL: activities of daily living; HRS: Health and Retirement Study; SHARE: Survey of Health, Ageing and Retirement in Europe; ELSA: English Longitudinal Study of Ageing; KLoSA: Korean Longitudinal Study of Ageing; MHAS: Mexican Health and Aging Study; LASI: Longitudinal Ageing Study in India; CHARLS: China Health and Retirement Longitudinal Study.

[a]BMI categories were classified as underweight (<18.5 kg/m^2), normal weight (≥18.5 kg/m^2 to <23 kg/m^2), overweight (≥23 kg/m^2 to <25 kg/m^2), and obesity (≥25 kg/m^2) in the KLoSA, LASI and CHARLS surveys.

[b]Limitations defined as reporting one or more limitations out of five items on the ADL scale.

[c] Logistic regression model adjusted for age, age^2, marital status, education, and prevalence of diabetes, arthritis, hypertension, stroke, cancer, lung disease, and heart disease.

p-values of Q test for differences in meta-analysis estimates between HIC and MIC.

Arrows denote estimates where the upper limit of the confidence interval exceeds the X-axis range.
